# A combination of genome-wide association and transcriptome analysis reveals candidate genes controlling harvest index-related traits in ***Brassica napus***

**DOI:** 10.1038/srep36452

**Published:** 2016-11-04

**Authors:** Kun Lu, Zhongchun Xiao, Hongju Jian, Liu Peng, Cunmin Qu, Minglian Fu, Bin He, Linmei Tie, Ying Liang, Xingfu Xu, Jiana Li

**Affiliations:** 1 College of Agronomy and Biotechnology, Southwest University, Beibei, Chongqing 400715, China; 2 Industrial Crops Institute, Yunnan Academy of Agricultural Sciences, Kunmimg 650205, China; 3 Agricultural Technology Extension Stationin Lincang City, Lincang 677000, China

## Abstract

Harvest index (HI), the ratio of seed mass to total biomass of the aboveground plant parts, is an important trait for harvestable yield of crops. Unfortunately, HI of *Brassica napus* is lower than that of other economically important crops. To identify candidate genes associated with high HI, a genome-wide association study of HI and four HI-related traits was conducted with 520 *B. napus* accessions cultivated in both Yunnan and Chongqing. We detected 294 single nucleotide polymorphisms significantly associated with the abovementioned traits, including 79 SNPs that affected two or more traits. Differentially expressed genes between extremely high- and low-HI accessions were identified in 8 tissues at two cultivated regions. Combination of linkage disequilibrium and transcriptome analyses revealed 33 functional candidate genes located within the confidence intervals of significant SNPs associated with more than one trait, such as *SHOOT GRAVITROPISM 5* (*Bna.SGR5*), *ATP-CITRATE LYASE A-3* (*Bna.ACLA-3*) and *CAROTENOID CLEAVAGE DIOXYGENASE 1* (*Bna.CCD1*), their orthologs in the *Arabidopsis thaliana* have been shown to play key roles in photosynthesis, inflorescence, and silique development. Our results provide insight into the molecular mechanisms underlying establishment of high-HI *B. napus* and lay a foundation for characterization of candidate genes aimed at developing high-HI *B. napus* varieties.

Harvest index (HI), also named the coefficient of economic yield, is the ratio of economic yield (seeds) to the total shoot dry matter, and represents a plant’s ability to convert photoassimilates into economic products. HI is closely associated with the transport and distribution of photosynthates, organ development, and plant architecture. Therefore, yield could be enhanced by increasing the HI of crops, or vise versa^1^.

Rapeseed (*Brassica napus*, AACC, *2n* = 38) is one of the most widely cultivated oilseed crops, worldwide. The global average yield of rapeseed production improved significantly, from 0.52 t/hm^2^ in 1961 to over 1.64 t/hm^2^ in 2007^2^, but the HI of rapeseed still remains much lower than that of other economically important crops, such as rice (*Oryza sativa*), wheat (*Triticum aestivum*), maize (*Zea may*), and soybean (*Glycine max*)^3^. HI is highly heritable, though it is still influenced by various environmental factors, crop photosynthetic characteristics, vascular properties, panicle traits, and nitrogen nutrition levels^1^. Therefore, it has been recognized as an important breeding target for yield improvement in *B. napus*.

Genome-wide association studies (GWASs) are a powerful approach for gaining insight into the genetic architecture of complex traits in many crops, and have been used to identify loci and candidate genes. In *B. napus*, GWASs have revealed genetic loci underlying traits of interest at relatively high resolution. Li *et al*.^4^ performed a GWAS of thousand seed weight (TSW) and related quality traits, and identified two single nucleotide polymorphisms (SNPs) significantly associated with TSW^4^. Eight quantitative trait loci (QTLs) associated with plant height and five associated with branch number were identified in a GWAS, based on the abovementioned genotype data^5^. Several candidate genes associated with seed germination and vigor were also identified in a GWAS analysis^6^. Recently, a GWAS of HI and HI-related traits of *B. napus* was carried out using a natural population of 155 *B. napus* cultivars^7^. The authors identified 9 significant SNPs associated with HI in the *B. napus* C subgenome, which explained 3.42% of the phenotypic variation^7^. Taken together, these findings illustrate the promise of GWAS for detecting QTLs that control important complex agronomic traits.

Identification of significant QTLs controlling HI and HI-related traits of *B. napus*, using GWAS, is a crucial step for further improvement of *B. napus* yield. To increase accuracy in QTL identification, and identify efficiently QTLs responsible for environmental variation, we cultivated 520 *B. napus* accessions at two extremely different environments in China, under the same cultivation regime. Linchang, Yunnan Province (YN) is a typical high-yield production region of *B. napus*. In this region, the average yield is over 5.3 tons per hectare^8^, whereas in Chongqing (CQ), the standard *B. napus* production region in the Yangtze River Basin, the yield is only about 2.7 tons per hectare^9^,^10^. Phenotypic data was collected and used to conduct GWAS for identification of significant SNPs controlling HI-related traits. Transcriptome analyses of 8 tissues from extremely high- and low-HI *B. napus* accessions were conducted to identify candidate genes within the confidence intervals of significant SNPs. These results not only provide insight into the genetic mechanisms underlying high-HI formation in *B. napus,* but lay a solid foundation for further functional validation of candidate genes and marker-based breeding of *B. napus* varieties for high HI.

## Results

### Variation in HI and four HI-related traits

HI and four HI-related traits (biomass yield (BY), canopy biomass yield (CBY), stem dry weight (ST), and seed yield (SY)) in the lines grown in three natural environments (CQ in 2013 (13CQ), CQ in 2014 (14CQ) and YN in 2014 (14YN)) and two virtual environments, (trait difference (14YN-CQ) and trait variation (14VTL) between YN and CQ in 2014) were analyzed using descriptive statistics and Analysis of Variance (ANOVA) (Table 1). Among the five investigated traits, the coefficient of variation (CV) of SY was higher than that of the other traits in most environments, reflecting large variation of SY within the entire GWAS panels. The average CV of HI was higher in the lines grown at YN than CQ. The CVs of different traits in 14VTL ranged from 18.98% to 48.31%, and those of SY and HI were highest, indicating that this trait was sensentive to environmental variation.

The five evaluated traits exhibited continuous variation in the accessions, both in CQ and YN (Supplementary Fig. S1), indicating that they were quantitative traits controlled by multiple genes. The estimate of broad-sense heritabilities ranged from 72.71% to 82.13%, suggesting that the majority of phenotypic variation for these traits, in *B. napus*, is attributed to genetic effects. In general, plants grown in the high-yield region had a larger ST and lower HI (Table 1), implying that photosynthetic products might be restrained in the stems of the accessions that have a lower transport efficiency. Hence, it should be possible to increase the *B. napus* yield and HI by increasing phloem transport of photosynthetic products from the stems to siliques.

### Population structure and model selection

**Figure 1:**
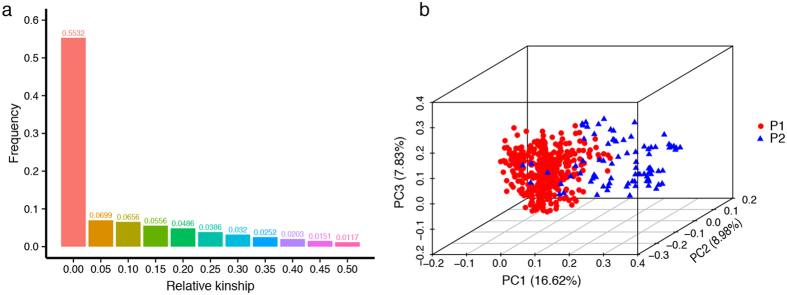
Distribution of pairwise relative kinship coefficients and PCA of 520 *B. napus* inbred lines. (**a**) Relative kinship coefficients and (**b**) PCA results. Only kinship coefficients of 0 to 0.5 are shown. Blue triangles and red circles represent the *B. napus* accessions in group 1 and group 2, respectively. Subpopulation in PCA plot analysis was assigned based on STRUCTURE analysis results from our previous study.

Based on a previous study, the 520 *B. napus* accessions were divided into two subgroups (P1 and P2)^11^ (Supplementary Table S1). We estimated the relative kinships^12^, and found 68.87% of the kinship coefficients ranged from 0 to 0.05, with 55.32% of pairwise kinship coefficients being 0 (Fig. 1a), indicating that most accessions have no, or only a weak genetic relationship with the other accessions and have only a small effect on the results of GWAS. Principal component analysis (PCA) demonstrated that the top two eigenvectors clearly separated these accessions into two subpopulations, which is consistent with the pattern of the genetic structure of the GWAS population (Fig. 1b).

Previous studies showed that kinship (K) and population structure (Q, PCA) can result in false associations in GWAS^13^. For all traits, we performed association analyses to evaluate the performance of six statistical models (i.e., the naive, Q, PCA, K, Q + K, and PCA + K models) (Supplementary Fig. S2 and Table 2). Generally, the observed -log_10_(*P*-value) values from the naive model greatly deviated from the expected distribution, followed by the K and Q models, whereas the PCA + K model showed a better effect than the other models for most traits, and consequently this model was used for association study in our experiment.

### Association mapping

**Figure 2:**
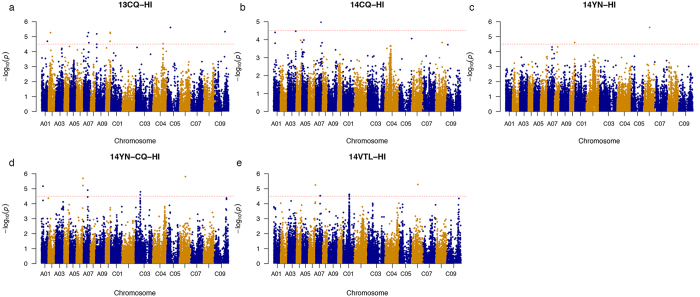
Manhattan plots of association analysis for HI under five environments. Manhattan plot of GWAS results for (**a**) 13CQ-HI, (**b**) 14CQ-HI, (**c**) 14YN-HI, (**d**) 14YN-CQ-HI, and (**e**) 14VTL-HI. The dashed horizontal red line depicts the significance threshold (−log_10_1/31,839 = 4.5).

Based on the optimal models, we detected 294 SNPs (*P* < 3.14E-05) significantly associated with HI and four HI-related traits (Fig. 2 and Supplementary Table S2), including 30, 26, 131, 88, and 19 SNPs associated with HI and with CBY, ST, BY, and SY, respectively. Under environment 13CQ, a total 250 significant SNPs were identified, followed by environments 14VTL, 14CQ, 14YN-CQ and 14YN, in which 18, 13, 9 and 4 significant SNPs, respectively, for different traits were detected.

Among these SNPs, 80 could be identified repeatedly, including 79 SNPs which associated with multiple traits (Fig. 2 and Supplementary Table S3). For example, 16 SNPs were simultaneously associated with BY, CBY, and ST. The marker Bn-A06-p23865356 was closely related to 14VTL-HI, 14VTL-SY, and 14YN-CQ-HI (Supplementary Fig. S3), whereas Bn-scaff_15763_1-p506136 on chromosome C06 was simultaneously associated with 14YN-HI, 14YN-ST, 14VTL-HI, and 14YN-CQ-HI (Supplementary Fig. S4). These significant SNPs associated with more than one trait were chosen for further candidate gene identification.

To identify the genotypes associated with relatively low biomass and high seed yield, genotypic classification analysis was performed by comparing the mean trait values between lines carrying different alleles of all significant SNPs. For most of the significant SNPs, higher seed yield of plants with favorable alleles possessed higher biomass yield (Supplementary Table S2). We also identified a few alleles of significant SNPs showing favorable effects on seed yield, but no effects on biomass yield at YN, such as Bn_scaff_19170_1_p1132242, Bn_scaff_19526_1_p67 and Bn_A09_p25738298 (Supplementary Fig. S5), which are interesting genotypes for further study.

### Identification of differentially expressed genes

To identify differentially expressed genes (DEGs) between extremely high- and low-HI *B. napus* lines at CQ and YN, 8 tissues harvested from four extremely high- and low-HI lines were separatively pooled as 32 independent samples (2 environments × 2 kinds of HI lines × 8 tissues). As a result, a total of 64 libraries (2 biological replicates per sample) were constructed and used for sequencing. After removing low quality and contaminant reads, a total of 1,385,050,482 125-bp clean paired-end reads were acquired, with an average of 21.64 million reads per sample, containing ~330 Gb of sequence data. On average, 83.62% of the input reads mapped uniquely to the *B. napus* reference genome^14^ (Supplementary Table S4).

**Figure 3:**
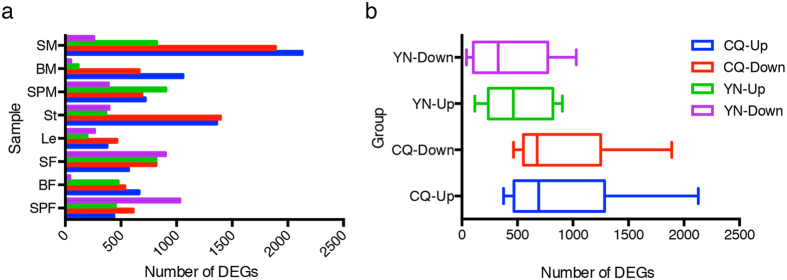
Comparison of DEGs between extremely high- and low-HI lines grown under two different environments. (**a**) Number of up- and down-regulated DEGs in 8 different tissues of plants grown at YN and CQ. (**b**) Average number of up- and down-regulated DEGs in all tissues of plants grown at YN and CQ. The “up” and “down” indicate up- and down-regulated genes, respectively.

RNA sequencing (RNA-seq) results showed that the number of DEGs was greatest in seeds harvested 20 days after flowering on the main inflorescence at CQ (2129 up-regulated and 1889 down-regulated genes), and on the primary branch at YN (816 up-regulated and 901 down-regulated genes) (Fig. 3a). The average number of DEGs between the high- and low-HI *B. napus* accessions grown at CQ (899 DEGs) was much higher than that in plants grown at YN (467 DEGs) (Fig. 3b), indicating that transcriptomic variation might be negatively correlated with yield in *B. napus*. At CQ, seeds on the main inflorescence (SM) showed the highest transcriptomic variation, whereas genes in seeds on the primary branch (SP) displayed highest transcriptomic variation at YN. Thus, stems and seeds appear to be important vegetative and reproductive tissues underlying differences in HI, since transcriptomic variation in these tissues was higher than in the other tissue types analysed.

### Gene Ontology (GO) and Kyoto Encyclopedia of Genes and Genomes (KEGG) enrichment analysis

**Figure 4:**
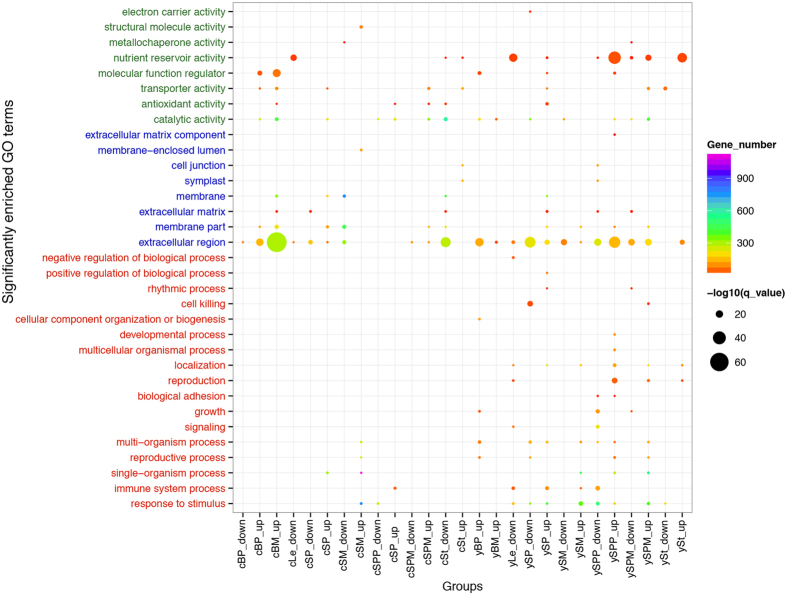
Significantly enriched level 2 GO terms of DEGs. First letters (c and y) in the group names represent the cultivation region (CQ and YN, respectively). The “up” and “down” indicate that the GO enrichment results were derived from up- and down-regulated genes, respectively.

GO enrichment analysis identified 3391 and 3704 significantly enriched GO terms from amongst the DEGs at CQ and YN, respectively (Supplementary Table S6). Among the significantly enriched level-2 GO terms (Fig. 4), nutrient reservoir activity (GO:0045735) was the GO term for Molecular Function with the highest overrepresentation (false discovery rate (FDR) = 6.31E-39). Extracellular region (GO:0005576) was the most overrepresented GO (FDR = 1.63E-65) among the GO terms for Cell Component. In addition, DEGs identified at YN showed higher enrichment for Biological Processes than those grown at CQ (Fig. 4). Cell wall organization or biogenesis (GO:0071554, FDR = 1.67E-48), photosynthetic membrane (GO:0034357, FDR = 2.57E-51), and enzyme regulator activity (GO:0030234, FDR = 1.21E-21) were the most enriched level-3 GO terms in Biological Process, Cell Component, and Molecular Function, respectively (Supplementary Figs S6, S7 and S8).

KEGG enrichment analysis identified 92 and 54 significantly enriched pathways associated with the up- and down-regulated genes, respectively (Supplementary Table S6). Among those significantly enriched pathways, flavonoid biosynthesis (ath00941), glucosinolate biosynthesis (ath00966), pentose and glucuronate interconversions (ath00040), phenylpropanoid biosynthesis (ath00940), starch and sucrose metabolism (ath00500), and cutin, suberine and wax biosynthesis (ath00073) were more abundant than other pathways, and significantly enriched in multiple samples. The ancient, conserved flavonoid biosynthesis and phenylpropanoid biosynthesis pathways might play critical roles in plant adaptation to environmental stresses, which seemed to be more important for *B. napus* cultivation at CQ. As the difference in HI among the *B. napus* accessions was mainly caused by differences in transport efficiency from source to sink organs, these DEGs over-represented in starch and sucrose metabolism might control the photoassimilate partitioning and allocation from the site of synthesis to regions of utilization and/or storage and underlie the variation in HI among the *B. napus* accessions.

### Identification of candidate genes

To identify candidate genes, we combined the GWAS and RNA-seq results, and only selected genes differentially expressed (DE) between the extremely high- and low-HI accessions, or encoded transcription factors (TFs) associated with high HI within the linkage disequilibrium (LD) blocks. Calculation of the LD blocks of 21 SNPs associated with more than one trait showed that 14 SNPs were located in LD blocks ranging in size from 231~498 kb, containing 35~128 genes (Supplementary Table S3). The 500-kb flanking sequences surrounding the remaining 7 significant SNPs were regarded as LD intervals.

**Figure 5:**
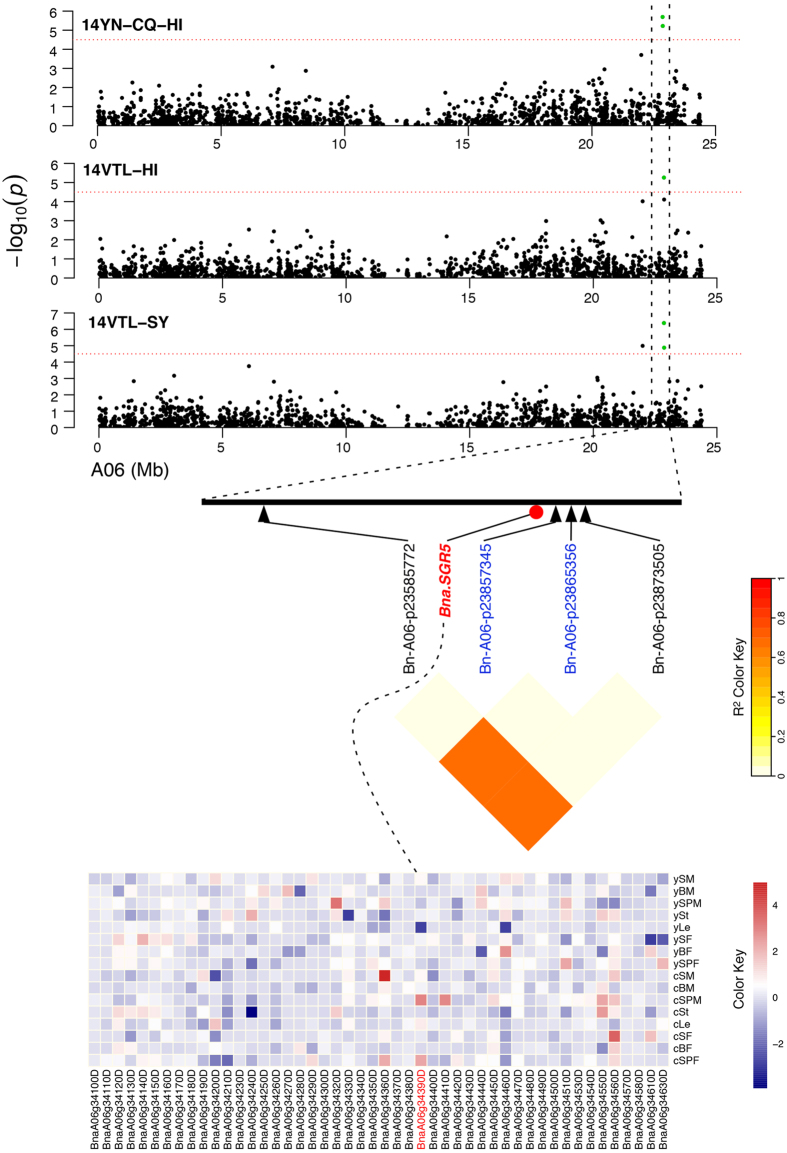
Candidate gene in the LD interval on chromosome A06 identified through GWAS. Top, the candidate gene region associated with HI and SY on chromosomes A06, as determined by GWAS. Middle, pairwise LD estimates in the haplotype block of the SNP marker Bn-A06-p23865356 on chromosome A06. Bottom, heatmap of candidate genes based on fold change of all genes in the haplotype block in 8 tissues of plants grown at YN and CQ. The dashed line represents the most important candidate gene in the LD heatmap and expression level.

In the 21 LD blocks, 73 genes encoding TFs and 174 DEGs were identified. The biological functions of the candidates were analyzed by performing Protein Basic Local Alignment Search Tool (BLASTP) searches against all *Arabidopsis* proteins. Of the 10 TFs that were DE within the LD intervals, 4 were regarded as important candidates. For example, *BnaA06g34390D* was located within the LD block of Bn-A06-p23865356 and encoded orthologs of *Arabidopsis SHOOT GRAVITROPISM 5* (*AtSGR5*) which is a regulator of auxin biosynthesis and transport and, thus, of the early events of gravitropism in inflorescence stems^15^ (Fig. 5). In addition, several DEGs were also chosen as their biological functions were critical for variation of HI, such as *BnaA02g13010D* and *BnaA02g12860D*, two genes located within the LD intervals of marker Bn-A02-p10067386, and encoded orthologs of TEOSINTE BRANCHED1, CYCLOIDEA, PCF1 (AtTCP1), CYTOCHROME P450, FAMILY 735, SUBFAMILY A, and POLYPEPTIDE 2 (AtCYP735A2) in *Arabidopsis*^16^; *BnaA03g38660D* was located 32 kb away from the marker Bn-A03-p20375514, and encoded an ortholog of *Arabidopsis* VACULOLAR SORTING RECEPTOR 3 (AtVSR3), which is essential for pollen tube growth^17^; *BnaA08g06910D* was 164 kb upstream of marker Bn-A08-p7814328, and encoded an ortholog of *Arabidopsis* HEAT SHOCK TRANSCRIPTION FACTOR A1D (AtHSFA1D), a TF known to confer thermotolerance in plants^18^. All candidate genes within the LD blocks associated with development and stress tolerance might contribute to the differences in HI and HI-related traits listed in Supplementary Table S3.

### Validation of DEGs by quantitative real-time polymerase chain reaction (qRT-PCR)

To validate the RNA-seq results, we selected 10 DEGs and evaluated their expression levels by qRT-PCR. All amplified PCR products were recovered for sequencing. The Pearson correlation coefficients between RNA-Seq and qRT-PCR data were calculated based on the log_2_ fold change. As shown in Supplementary Figs S9 and S10, the expression patterns of DEGs detected by qRT-PCR showed significant similarity (*r*^2^ = 0.9322) with the RNA-Seq data, confirming the reliability and accuracy of the RNA-seq data.

## Discussion

HI is a complex agronomic trait that is influenced by a variety of physiological and environmental factors, and varies widely among crops. For modern varieties of the most intensively-cultivated grain crops, including rice^19^, wheat^20^, barley (*Hordeum vulgare*)^21^, and maize^22^, HI values range from 0.4 to 0.6^3^,^23^. Genetic and agronomic improvements have greatly increased the yield potential of *Glycine max* (soybean), which is associated with an increase in HI from around 0.35 to 0.53^3^. According to previous reports, the average HI for *B. napus* was 0.28 (*n* = 117)^24^, only higher than that of oat (0.21), with values ranging from 0.04 to 0.41^25^. In our study, the HI ranged from 0.048 to 0.42 and 0.052 to 0.34 for plants grown at CQ and YN, respectively, a finding consistent with earlier studies. although the HI was relatively stable in *B. napus*, and the observed variation was less than that of most other field crops examined^24^, some *B. napus* accesscions with high HI ( > 0.4) were identified, suggesting that the potential for futher yield increase in *B. napus*.

Correlation analyses showed that SY was significantly and positively related to HI (Supplementary Table S6), which is in line with the findings of several previous studies^7^,^26^. In another important crop, soybean, yield increase was also positively associated with HI^27^. These results suggest that yield can be enhanced in *B. napus* by increasing HI at both high-yield and standard production regions in China.

In the present study, ST was not correlated with HI in plants grown at CQ in 2013 and 2014, and tended to be negatively associated with HI in those grown at YN in 2014. The stem is an important organ in *B. napus*, accumulating large amount of stored photosynthate and nitrogen during the vegetative stage, and reallocating these assimilates and most nitrogen to seeds during the filling stage^28^. The significant negative correlation observed between the ST and HI indicates that phloem translocation of photoassimilates and their partitioning to seeds is a key factor limiting yield improvement under high-yield growth conditions. A similar phenomenon was observed in hybrid rice, namely that poor translocation and partitioning of assimilates to the grain resulted in limited grain filling and a low HI^29^. In addition, there was a significant positive relationship between HI and CBY.

In the Brassicaceae, silique walls are the major photosynthetically active organ after flowering and contribute assimilates and nutrients to fill seed growth^30^. Plants with higher CBY may be expected to possess more siliques, and thus to produce more assimilates and have a higher SY and HI. Furthermore, we noticed that the relationship between BY and HI was different in plants grown at CQ and YN. A similar inconsistency was observed in soybean grown in different environments^27^, indicating that HI is more complex than other HI-related traits, and might be easily affected by differences in cultivation environments. In addition, CBY and BY were both significantly positively related to SY, which is consistent with previous evidence^31^, suggesting yield potential greatly depends on biomass accumulation in *B. napus*.

In GWASs, general linear models (GLMs) often lead to false positives (type I errors), whereas mixed linear models (MLMs) are more stringent and can result in false negatives (type II errors)^32^. The PCA model has been widely used to correct for population stratification in GWASs^33^ and after integrating the kinship, this mixed PCA + K model gave significant improvement in reducing spurious associations, and thus has been broadly used in GWASs of several crops^4^. In the current study, six models, involving different combinations of K, Q, and PCA were used to analyze all of the traits examined this our study. As with early studies, the mixed PCA + K model was best in removing false-positive association signals (Table 2), indicating that population structure correction in a PCA model was more efficient than the STRUCTURE algorithm at eliminating false positives^34^. Hence, to reduce the number of false positives and negatives, only optimal models for each trait were considered when identifying association signals.

Over the past several years, several QTLs for important traits in *B. napus* were identified in GWAS, providing relatively higher mapping resolution and more candidate loci than in studies based on biparental populations^35^. In our study, a large natural population composed of 520 *B. napus* accessions was phenotyped by the 60K *Brassica* SNP array. GWAS results showed that the most significant SNPs for HI in plants grown at CQ and YN, in 2014, were located on chromosomes C05 and A07, and explained 5.66% and 4.76% of the phenotypic variation, respectively. For BY, a previous GWAS only detected a single significant SNP, which explained 0.76% of phenotypic variation^7^, whereas the most significant SNP we identified on chromosome A03, in our GWAS results from 13CQ, explained 8.36% of phenotypic variation. Arguable, this identified higher contribution rate could well be related to the larger population size employed in our GWAS. Consistent with this notion, rice scientists have perfomed whole-genome resequencing on 3000 rice accessions to obtain a global representation of genetic and functional diversity^36^.

Recently, Körber *et al*.^37^ idenfitifed only two significantly associated SNPs for SY due to the fact that this trait was examined at only two locations^37^, and Zhao *et al*.^31^ detected 9 consensus QTLs for SY, mainly located on chromosomes A02, A06, C01, and C06^31^. In the present study, significantly associated SNPs Bn-A02-p10067386 and Bn-scaff_16547_1-p307962, located on chromosomes A02 and C06, respectively, were closely co-localized with previously identified QTLs for SY, indicating that these QTLs were stable and could be repeatedly detected in different environments, and thus suitable for marker-assisted selection in high-yield breeding of *B. napus*.

The average yield of *B. napus* cultivated at YN is much higher than that in standard production regions. To elucidate how the environment affects HI and HI-related traits, we performed GWAS in virtual environments 14VTL and 14YN-CQ, to identify the significantly associated signals for each trait responsible for environmental change. We detected 8 and 7 significant SNPs for HI in environments 14VTL and 14YN-CQ, respectively, and of these 3 could be found repeatedly in the two environments. In addition, Bn-scaff_15763_1-p506136 was significantly associated with HI in both 14YN and 14YN-CQ environments. These results indicate that these associated signals were stable, and this method would be a very useful strategy for identifying loci that control plant responses to environmental variation.

Combining GWAS and RNA-seq can provide both the power and resolution needed to identify DE candidate genes, and has proven to be more successful than either strategy alone^38^,^39^. Based on integrative analyses of RNA-seq and GWAS in *B. napus*, 8 and 24 candidate genes putatively associated with drought tolerance and stem resistance to *Sclerotinia sclerotiorum*, respectively, were identified^38^,^40^. More recently, QTL fine mapping, digital gene expression (DGE) profiling, and whole-genome re-sequencing (WGS) were combined to identify a *NODULIN 26-LIKE INTRINSIC PROTEIN* gene involved in regulating boron uptake efficiency in *B. napus*^39^. Applying a similar strategy, we identified 73 TFs and 174 genes that were DE in at least one tissue from a total of 1113 genes within the LD blocks of 21 significant SNPs that associated with more than one trait. Taken together, these findings demonstrate the efficacy of combining strategies for rapid identification of candidate genes associated with complex traits.

The SNP Bn-A02-p10067386, associated with both HI and SY, is of particular interest. Within the LD block, *BnaA02g12860D* encodes an ortholog of AtCYP735A2, which is a cytochrome P450 monooxygenase that catalyzes the *trans*-hydroxylation of cytokinins (CKs) to form *trans*-zeatin (tZ)-type CK^41^. In *Arabidopsis* this gene defines the size of rosette leaves, inflorescence stems, and the number of flowers and siliques. We note that expression of *BnaA02g12860D* was significantly up-regulated in the leaf (Le) and silique pericarps, located on the main inflorescence (SPM) of the high HI accessions grown at CQ, implying that this gene might encode a regulator of HI and SY.

The *BnaA03g38390D* gene, located within the LD block of Bn-A03-p20375514 associated with BY, CBY, and ST at 13CQ, encodes an ortholog of the nuclear transcription factor Y, subunit B 7 (AtNF-YB7)^42^. Overexpression of *AtNF-YB7* resulted in earlier seedling establishment and higher biomass than controls through an increase in carbon assimilation and a reduction in transpiration. In this regard, *BnaA03g38390D* showed significantly higher expression in BM of the high HI accessions at CQ. Hence, the role of this gene in shoot development of the high HI accessions deserves further study.

We identified *BnaA06g34390D* within the LD block of the Bn-A06-p23865356, and established that it encoded an ortholog of *AtSGR5* (Fig. 5). As a C2H2-type TF, *AtSGR5* is involved in the early events of gravitropism in the inflorescence stem^15^. Up-regulation of *BnaA06g34390D* expression was detected in the SPM and silique pericarps on the primary branch (denoted as SPP) at CQ, implying that seed yield at CQ and HI might be affected by the angle of the SP, becasue silique wall photosynthesis is known to be important for seed filling. Further functional characterization of *BnaA06g34390D* is needed to reveal the physiological significance of the involvement of this gene in the variation of HI in *B. napus*. It is notable that in this LD bock, three significant SNPs were detected specifically in the virtual environments, which were regarded as candidate loci for HI and SY associated with environment variations, implying environment-specific effects on HI and SY.

Within the LD interval of the Bn-A08-p20354609, *BnaA08g26390D* was identified as the most plausible candidate gene, and it encodes an ortholog of ATP-CITRATE LYASE A-3 (ACLA-3) in *Arabidopsis.* Importantly, this gene was significantly up-regulated in all 8 tissues in plants grown at YN and CQ. A previous study showed that plants with reduced ACL activity have a complex bonsai phenotype, with features such as miniaturized organs, smaller cells, aberrant plastid morphology, and reduced cuticular wax deposition^43^. Hence, the significantly higher expression of *BnaA08g26390D* in vegetative tissues might be important for accumulating more photosynthates in the higher HI *B. napus* accessions.

**Figure 6:**
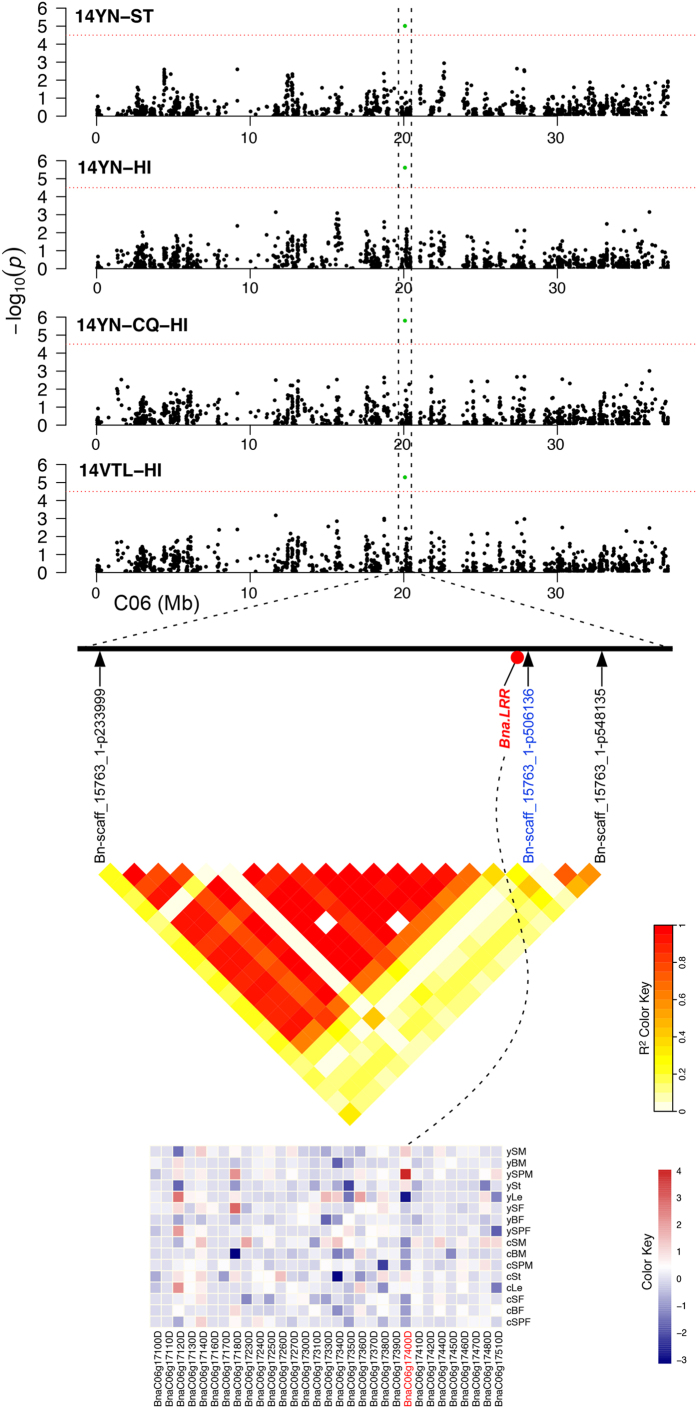
Candidate gene in the LD interval on chromosome C06 identified through GWAS. Top, the candidate gene region associated with HI and ST on chromosome C06, as determined by GWAS. Middle, pairwise LD estimates in the haplotype block of the SNP marker Bn-scaff_15763_1-p506136 on chromosome C06. Bottom, heatmap of candidate genes based on fold change of all the genes in the haplotype block in 8 tissues of plants grown at both YN and CQ. The dashed line represents the candidate gene associated with high HI in the LD heatmap and expression level.

The LD block of the Bn-scaff_15763_1-p506136 is another noteworthy region, as here, a gene encoding a leucine-rich repeat (LRR) family protein, *BnaC06g17400D* was significantly up-regulated in seeds of the high HI *B. napus* accessions grown at YN, but repressed in plants grown at CQ (Fig. 6). Functional analysis of BnaC06g17400D on STRING (Search Tool for the Retrieval of Interacting Genes/Proteins, http://string-db.org)^44^ revealed that the protein was the ortholog of AT3G59510 (Leucine-rich repeat (LRR) family protein), and interacted with AT4G24190 (HEAT SHOCK PROTEIN 90.7), which encodes an endoplasmic reticulum (ER)-resident HSP90-like protein and is required for shoot apical meristem, root apical meristem, and floral meristem formation and pollen tube elongation^45^. Hence, we propose that BnaC06g17400D is also a key regulator of high HI formation at both CQ and YN.

Within the confidence interval of Bn-scaff_16197_1-p1970599, *BnaC08g33690D* encodes CAROTENOID CLEAVAGE DIOXYGENASE 1 (CCD1), which plays essential roles in photosynthesis, plant development, and abiotic stress adaptation^46^. The expression levels of those candidate genes were significantly up-regulated in vegetative tissues in plants grown at CQ and YN. Thus, these genes should also be considered as important candidates for HI variation.

In this study, we identified several significant SNPs and candidate genes associated with HI and HI-related traits. However, we realized that the accuracy of our GWAS results is impacted, especially for such complex traits, because only representative environments were chosen for field trials. Hence, further studies are needed to validate repeatability of these significant SNPs, under multiple environments, and biological functions for these candidate genes will need to be experimentally verified.

In summary, our study demonstrated that HI is a complex agronomic trait that is influenced by both environmental and genotypic factors. The substantial phenotypic variation for HI and HI-related traits in *B. napus*, at both high-yield and standard production regions provides an excellent opportunity to dissect the genetic architecture of these complex traits, with a focus on the effect of environmental variation. Using a combination of GWAS and RNA-seq, we identified a large number of novel loci associated with HI, and predicted 33 candidate genes. These genes can be used in marker-based breeding programmes for increased HI.

## Methods

### Plant materials and field trials

The natural population of *B. napus* provided by the Chongqing Rapeseed Engineering Research Center, China, consisted of 520 genetically diverse inbred lines (Supplementary Table S1). Most of these accessions were derived from research institutions in China, but several were introduced from other countries, including Germany, Australia, and Canada.

All rapeseed inbred lines were planted, in triplicate, in a randomized block design. Each plot contained three rows; 10 plants per row, 20 cm between plants within each row, and 30 cm between rows. Plants were cultivated under natural environments, as follows: 520 accessions were cultivated in Beibei, Chonqging (29°45′ N, 106°22′ E, 238.57 m, CQ), for two consecutive years (2012–13 and 2013–14, denoted as 13CQ and 14CQ) and, based on the flowering time observed in 2013 at CQ, 320 accessions were selected for planting at Lincang, Yunnan Province (23°43′ N, 100°02′ E, 1819.50 m, YN) in 2014 (14YN). All accessions were sown at the end of September in both YN and CQ, and harvested at the beginning of May (CQ) or the end of April (YN) in the following year.

After harvesting, plants were divided into canopy and stem, and dried at 50 °C to a constant weight in a ventilated drying oven. Canopy biomass yield (CBY) and stem dry weight (ST) were defined as the dried weight of canopy and stems, respectively. Biomass yield (BY) was represented as the sum of CBY and ST. Seed yield,per plant (SY), was determined by measuring the dry weight of all seeds produced per plant. Harvest index (HI) was represented as the ratio of SY to BY.

To identify the significant associated signals for each trait responsible for environmental variation, two virtual environments were derived from the abovementioned five environments. The first virtual environment was the trait difference between YN and CQ in 2014 (denoted as 14YN-CQ) and the second virtual environment was the trait variation between two the locations in 2014 (denoted as 14VTL), which was calculated by the following formula: 2 × (YN − CQ)/(CQ + YN). The mean value of the ten plants with the same height in the same plot represents the trait value of a replication, and the mean value of the three replications was regarded as the phenotypic value of the target trait.

### Statistical analysis

The ANOVA was conducted using GLM procedure in SAS version 8.0 (SAS Institute Inc.), with the following model: *y*_*ger*_ = *μ* + *α*_*g*_ + *β*_*e*_ + (*αβ*)_*ge*_ + *γ*_*r*(*e*)_ + *ε*_*ger*_, where *y*_*ger*_ was the phenotypic trait value of the *g*^th^ genotype in the *e*^th^ environment for the *r*^th^ replicate, *μ* the grand mean, *α*_*g*_ the genotypic effect of the *g*^th^ genotype, *β*_*e*_ the effect of the *e*^th^ environment, (*αβ*)_*ge*_ the interaction effect of the *g*^th^ genotype and the *e*^th^ environment, *γ*_*r*(*e*)_ the block effect within the *e*^th^ environment, and *ε*_*ger*_ the residual. All effects were modeled as random. The CORR procedure in SAS version 8.0 (SAS Institute Inc.) was used to calculate the Pearson’s correlation coefficients between traits of interest. The broad-sense heritability was calculated as: 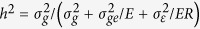, where 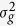, 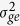 and 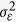 are estimates of the variances of genotype, genotype × environment interactions, and error, respectively. *E* is the number of environments, and *R* is the number of replications per environment.

### Genotyping analysis

Genotyping of the 520 *B. napus* accessions was carried out in Huazhong Agricultural University, Wuhan, China, using the *Brassica* 60 K Illumina Infinium SNP array. The SNP data were analyzed by Illumina BeadStudio genotyping software, as previously described^11^. SNPs with minor allele frequencies (MAFs) of <0.05, or lacking a call higher than 0.9 were excluded from analysis. After processing, 31,839 SNP markers were retained for further analysis. To determine the physical localization of SNPs, flanking sequences were subjected to BLASTN searches against the *B. napus* genome, with an E-value cut-off of 1E-5^14^,^47^. SNPs with a maximum Bit-Score were retained as unique SNPs, and subjected to further analysis.

### Population structure and genome-wide association analysis

The population structure (Q) was inferred using STRUCTURE 2.3.4, as we previously reported^11^. The relative kinship matrix (K) and principal component (PC) of the natural population were calculated using TASSEL 5.2.1^12^. Linkage disequilibrium (LD) between all pairs of SNP markers was estimated as the correlation of allele frequencies (*r*^2^) using TASSEL 5.2.1.

The effects of population structure (Q, PCA) and kinship (K) were estimated by a GWAS using the following six models: (a) naive model, which does not control for Q and K; (b) Q model, which controls for the percentage of admixture (Q matrix); (c) PCA model, which uses the top 10 PCs as fixed effects; (d) K model, which controls for K; (e) Q + K model, which uses both K as a random effect and the Q matrix; and (f) P + K model, which controls for both PCA and K. The former three models were performed using a GLM, while the remaining models were performed using an MLM in TASSEL 5.2.1^12^. The optimal model for each trait was selected based on a Quantile-quantile (Q-Q) plot in ggplot2^48^, by plotting the −log_10_(*p*) values of each SNP against the expected value. Suggestive (1/*n*) *P*-value threshold was set to control the genome-wide type 1 error rate, and *n* represented the number of SNP markers. The Manhattan plot was produced by qqman software^49^.

### RNA-seq and identification of differentially expressed genes

To identify differentially expressed genes (DEGs) between extremely high and low HI lines, total RNA was pooled at equal molar amounts from four extremely high and low HI *B. napus* lines (Supplementary Table S7). For each type of line, eight tissues, including mature leaves (Le) and stems (St), and buds on the primary branch (BP) and the main inflorescence (BM), were harvested at flowering stage 63–65, according to the BBCH (Biologische Bundesanstalt, Bundessortenamt and CHemical industry) scale^50^. Seeds and silique pericarps on the main inflorescence and the primary branch were harvested at 20 days after flowering. For each sample, two biological replicates, each harvested from three independent plants, were collected for RNA-seq and qRT-PCR analysis.

Sequencing library preparation and sequencing reactions were conducted at the Biomarker Technologies Corporation (Beijing, China). RNA-seq data were deposited in the NCBI under the accession number SRP072900. All sequencing reads were subjected to quality filtering using Trimmomatic-0.33^51^, and mapped to the *B. napus* reference genome using STAR 2.4.2a with default parameters. Gene expression levels were quantified by the cuffquant program in terms of fragments per kilobase of exon per million mapped fragments (FPKM)^52^. The cuffdiff program was used to test for statistically significant differences in gene expression between two samples. DEGs were identified based on the following criteria: false discovery rate (FDR) <0.05 and absolute fold change >2^53^. To identify and classify transcription factors (TFs) in *B. napus*, the putative amino acids were aligned with *Arabidopsis* TFs downloaded from the Plant Transcription Factor Database v3.0 (http://planttfdb.cbi.pku.edu.cn) and The Arabidopsis Information Resource (http://www.arabidopsis.org)^54^.

### GO and KEGG enrichment analyses of DEGs

To determine the biological significance of DEGs, all *B. napus* genes were annotated by BLASTP analysis against the *Arabidopsis* proteome (TAIR10) with an E-value cut-off of 1E-5^46^. GO enrichment analysis was implemented using BinGO v2.4.4, and significantly enriched GO terms (FDR <0.05) were displayed using ggplot2^48^,^55^,^56^. Kyoto Encyclopedia of Genes and Genomes (KEGG) pathway enrichment analysis was conducted on the KOBAS 2.0^57^. All GO and KEGG statistical tests were corrected for multiple comparisons (Benjamini Hochberg method).

### Identification of candidate genes

To define regions of interest for identification of candidate genes, the haplotype blocks were investigated with Haploview 4.2^58^, and the LD blocks were defined by the Four Gamete Rule with a fourth haplotype frequency cutoff of 0.1. All genes within the LD block were considered for identification of candidates. For significantly associated markers outside of the LD blocks, the 250-kb flanking regions on either side of the markers were used to identify candidates. Differentially expressed (DE) candidate genes were identified by screening the DEGs within the LD blocks or flanking regions of significantally associated markers.

### Validation of DEGs by qRT-PCR

Ten DE candidate genes identified in the abovementioned analyses were assayed by qRT-PCR. Gene-specific primers were designed using Geneious Pro 8.1.5^59^ (Supplementary Table S8). The total RNA was the same as used for RNA sequencing. cDNA synthesis, qRT-PCR cycling conditions, amplification efficiency, and specificity assessment were as previously described^60^. Two independent biological replicates, each with three technical replicates were analyzed per sample. Relative expression levels were calculated using the 2^*−ΔΔ*^Ct method, with *Bna.ACT7* and *Bna.UBC21* as internal controls^61^.

## Additional Information

**How to cite this article**: Lu, K. *et al*. A combination of genome-wide association and transcriptome analysis reveals candidate genes controlling harvest index-related traits in *Brassica napus*. *Sci. Rep.* **6**, 36452; doi: 10.1038/srep36452 (2016).

**Publisher’s note:** Springer Nature remains neutral with regard to jurisdictional claims in published maps and institutional affiliations.

## Supplementary Material

Supplementary Information

## Figures and Tables

**Table 1:** Mean values and heritability of HI and four HI-related traits.

Traita	Env.^b^	Number of accessions Tested	Mean ± SD	Range	CV (%)	*h*^2^ (%)
CBY	13CQ	476	66.17 ± 15.1	22.38~129.63	22.82	74.71
	14CQ	516	52.85 ± 14.04	16.32~94.27	26.57	
	14YN	302	107.67 ± 21.08	49.00~177.5	19.57	
	14YN-CQ	300	52.47 ± 23.44	−31.37~112.3	29.21	
	14VTL	300	0.64 ± 0.27	−0.43~1.41		
ST	13CQ	476	26.79 ± 6.8	9.50~54.88	25.53	82.13
	14CQ	516	37.41 ± 9.56	13.99~69.63	25.55	
	14YN	302	58.31 ± 15.51	27.67~128.67	26.61	
	14YN-CQ	300	19.39 ± 15.18	−20.62~74.37	31.72	
	14VTL	300	0.38 ± 0.27	−0.43~1.12		
BY	13CQ	476	92.97 ± 20.5	36.31~184.50	22.05	72.71
	14CQ	515	90.29 ± 21.65	37.33~159.00	23.98	
	14YN	302	165.56 ± 31.51	76.67~265.5	19.03	
	14YN-CQ	300	71.39 ± 34.49	−37.82~167.95	26.96	
	14VTL	300	0.54 ± 0.25	−0.33~1.21		
SY	13CQ	496	22.92 ± 7.8	1.46~53.39	33.98	77.04
	14CQ	516	18.10 ± 6.18	2.82~40.85	34.14	
	14YN	302	32.15 ± 10.74	6.34~61.07	33.41	
	14YN-CQ	300	12.77 ± 12.14	−34.51~49.56	48.31	
	14VTL	300	0.46 ± 0.46	−1.46~1.60		
HI	13CQ	476	0.23 ± 0.05	4.8%~42%	22.00	75.20
	14CQ	514	0.20 ± 0.042	4.5%~33%	21.00	
	14YN	302	0.19 ± 0.054	5.2%~31%	28.20	
	14YN-CQ	300	−0.01 ± 0.07	−0.21~0.16		
	14VTL	300	−0.08 ± 0.37	−1.33~0.90		

^a^CBY, canopy biomass yield; ST, stem dry weight; BY, biomass yield; SY, seed yield per plant.

^b^Environments: 13CQ, CQ in 2013; 14CQ, CQ in 2014; 14YN, YN in 2014; 14YN−CQ: 14YN−14CQ 14VTL, 2 × (14YN−14CQ)/(14YN + 14CQ).

SD, standard deviation; CV(%), coefficient of variation. *h*^2^, broad-sense heritability.

**Table 2:** Summary of significantly associated SNPs for HI and four HI-related traits.

**Trait**	**Env.**	**NSS**^**c**^	**Chromosome**	***R***^**2 d**^
CBY	13CQ	22	A03/A06/A07/A08/A09/C03/C05/C07/C08	4.37%~5.96%
	14CQ	2	A03/A06	3.71%~5.24%
	14YN	0		
	14YN-CQ	1	C01	8.24%
	14VTL	1	A08	8.09%
ST	13CQ	123	A08/A03	4.54%~9.69%
	14CQ	3	A03	4.51%~6.43%
	14YN	2	A03/C06	7.31%~8.51%
	14YN-CQ	0		
	14VTL	3	A08/A10	7.81%~8.13%
BY	13CQ	83	A03/A04/A05/A06/A07/A08/A09/C01/C02/C03/C04/C05/C07/C08/C09	4.56%~8.36%
	14CQ	5	A03/A09/C02/C05	3.47%~4.53%
	14YN	0		
	14YN-CQ	0		
	14VTL	0		
SY	13CQ	10	A02/A03/A05/A09/C01/C05/C06/C08	5.38%~7.49%
	14CQ	2	A03/C04	4.24%~4.25%
	14YN	0		
	14YN-CQ	1	A04	8.12%
	14VTL	6	A03/A04/A06	7.96%~11.01%
HI	13CQ	12	A01/A02/A07/A09/A10/C04/C05/C09	4.63–5.66%
	14CQ	1	A07	4.76%
	14YN	2	A10/C06	7.86%~8.51%
	14YN-CQ	7	A01/A06/A07/C03/C06	7.43%~9.61%
	14VTL	8	A06/A07/C01/C06	5.51%~8.68%

NSS, number of significantly associated SNPs; R2, phenotypic variance.
