# Supplementary material for: A combination of genome-wide association and transcriptome analysis reveals candidate genes controlling harvest index-related traits in Brassica napus
Source: Sci Rep. 2016 Nov 4;6:36452. doi: 10.1038/srep36452 (PMC5095561; doi:10.1038/srep36452)
Supplement: Supplementary Information [file srep36452-s1.pdf]

A combination of genome-wide association and transcriptome analysis reveals candidate genes controlling harvest index-related traits in *Brassica napus*

Kun Lu[1, †], Zhongchun Xiao[1, †], Hongju Jian[1, †], Liu Peng[1], Cunming Qu[1], Minglian Fu[2], Bin He[3], Linmei Tie[2], Ying Liang[1], Xingfu Xu[1], Jiana Li[1,*]

[1] College of Agronomy and Biotechnology, Southwest University, Beibei, Chongqing 400715, China

2 Industrial Crops Institute, Yunnan Academy of Agricultural Sciences, Kunmimg 650205, China

[3] Agricultural Technology Extension Stationin Lincang City, Lincang 677000, China

[†] These authors contributed equally to this work

[*] Corresponding author

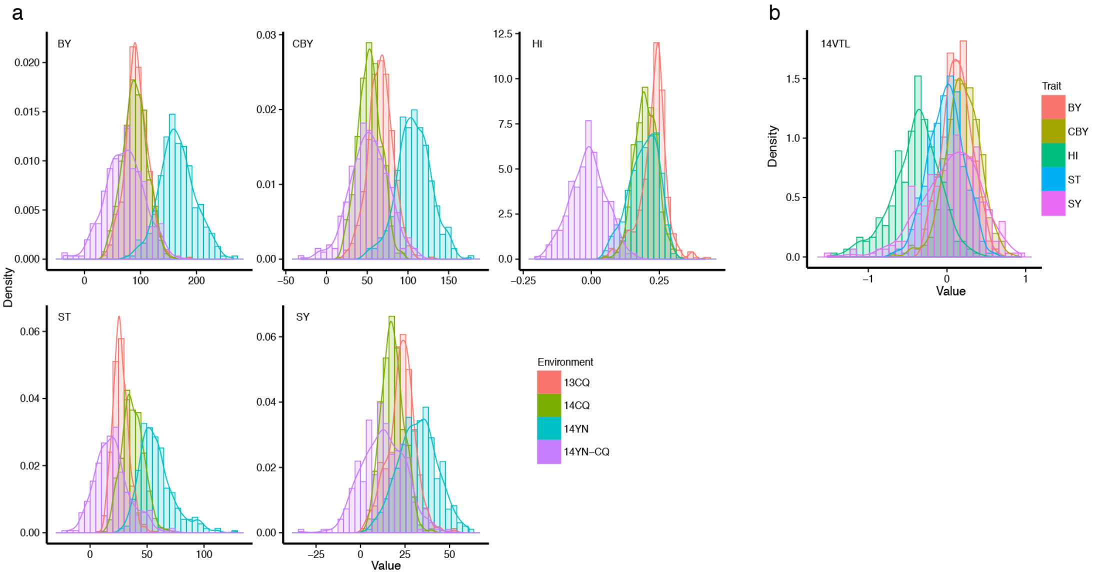

Supplementary Figure S1. Frequency distribution of HI and four HI-related traits in lines grown at CQ and YN. (a) Density of HI and four HI-related traits under environments 13CQ, 14CQ, 14YN, and 14YN-CQ, and (b) Density of HI and four HI-related traits under environment 14VTL. CBY, canopy biomass yield; ST, stem dry weight; BY, biomass yield; SY, seed yield. Environments: 13CQ, Chongqing in 2013; 14CQ, Chongqing in 2014; 14YN, Yunnan in 2014; 14YN−CQ, 14YN – 14CQ. 14VTL, 2× (14YN – 14CQ) / (14YN + 14 CQ).

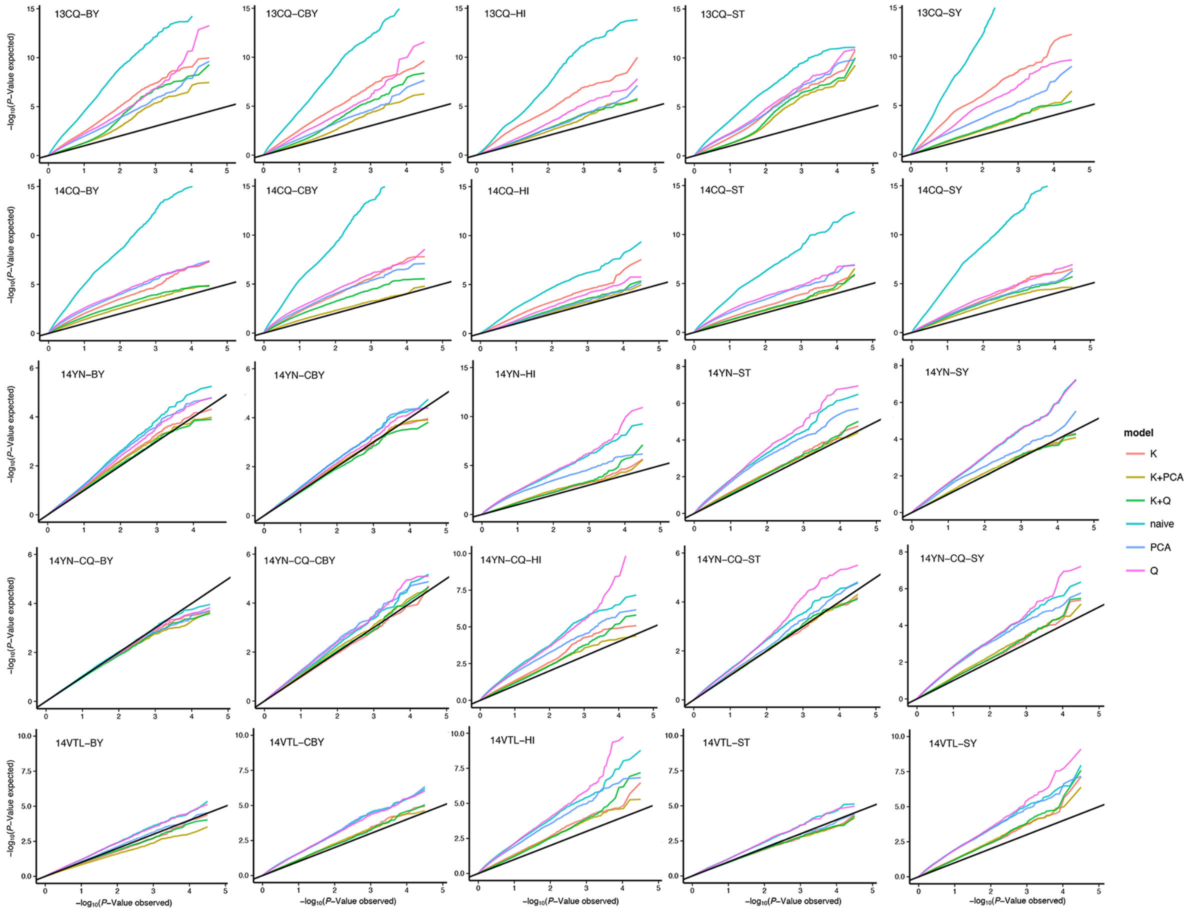

Supplementary Figure S2. Quantile-quantile plots for five traits in lines grown in five different environments. CBY, canopy biomass yield; ST, stem dry weight; BY, biomass yield; SY, seed yield per plant. Environments: 13CQ, Chongqing in 2013; 14CQ, Chongqing in 2014; 14YN, Yunnan in 2014; 14YN−CQ, 14YN – 14CQ; 14VTL, 2× (14YN – 14CQ) / (14YN + 14 CQ). Distribution of *P*-values assuming associations (expected *P*-values) are represented as black lines; distribution of *P*-values calculated based on the six models (observed *P*-values) are represented as different colored lines.

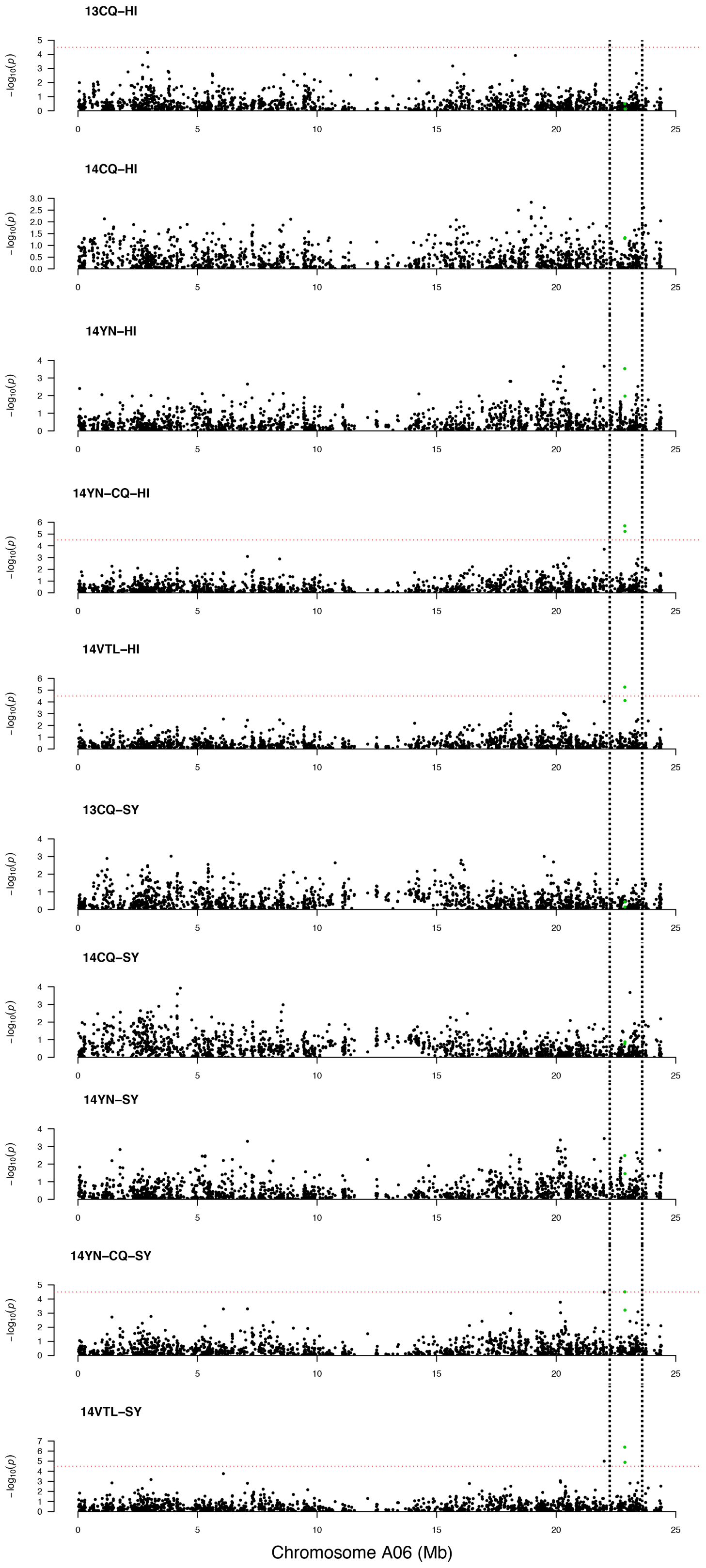

Supplementary Figure S3. Manhattan plot of the GWAS results of HI and SY traits on chromosome A06. SNPs of interest are shown in green dots, which located between two vertical dashed lines.

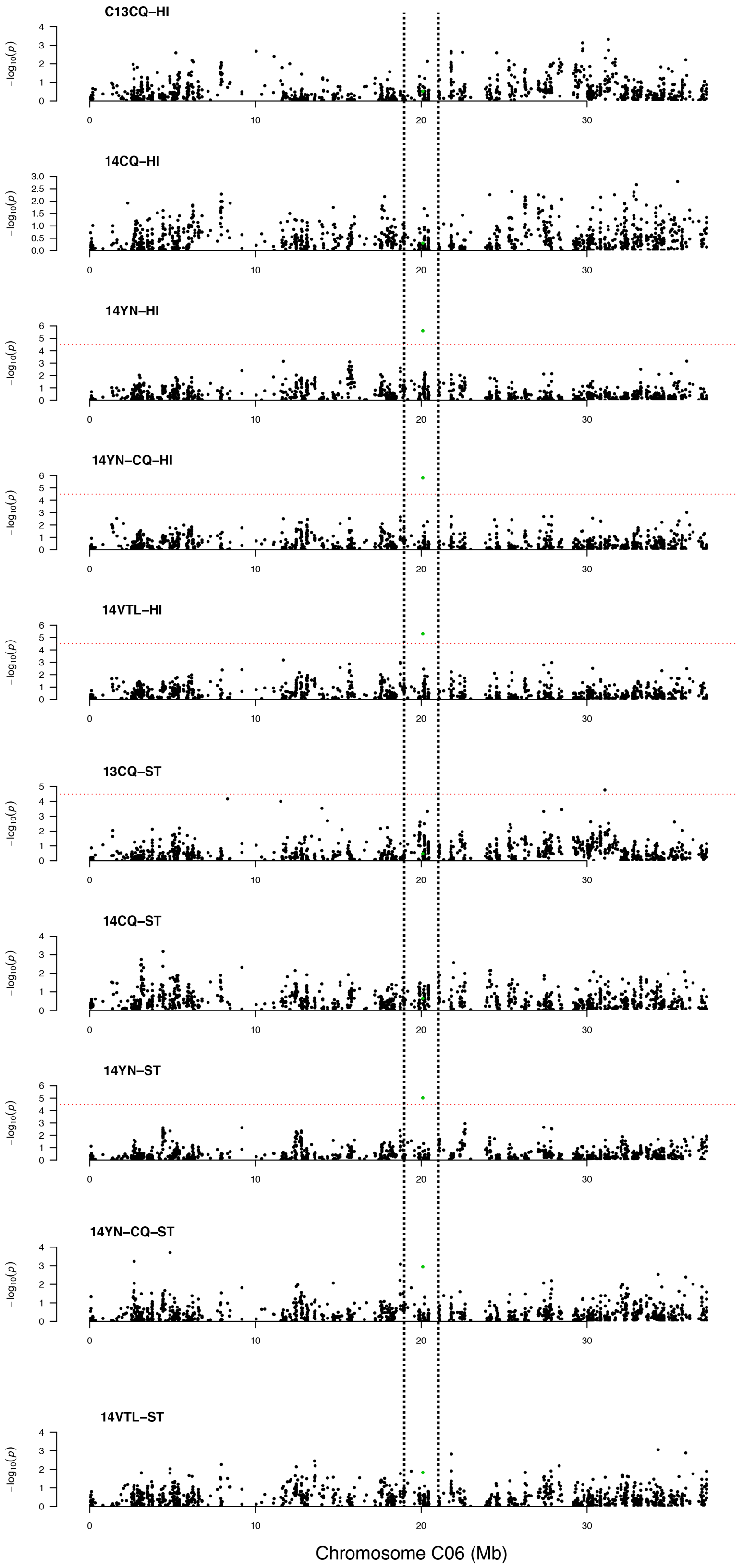

Supplementary Figure S4. Manhattan plot of the GWAS results of HI and ST traits on chromosome C06. SNPs of interest are shown in green dots, which located between two vertical dashed lines.

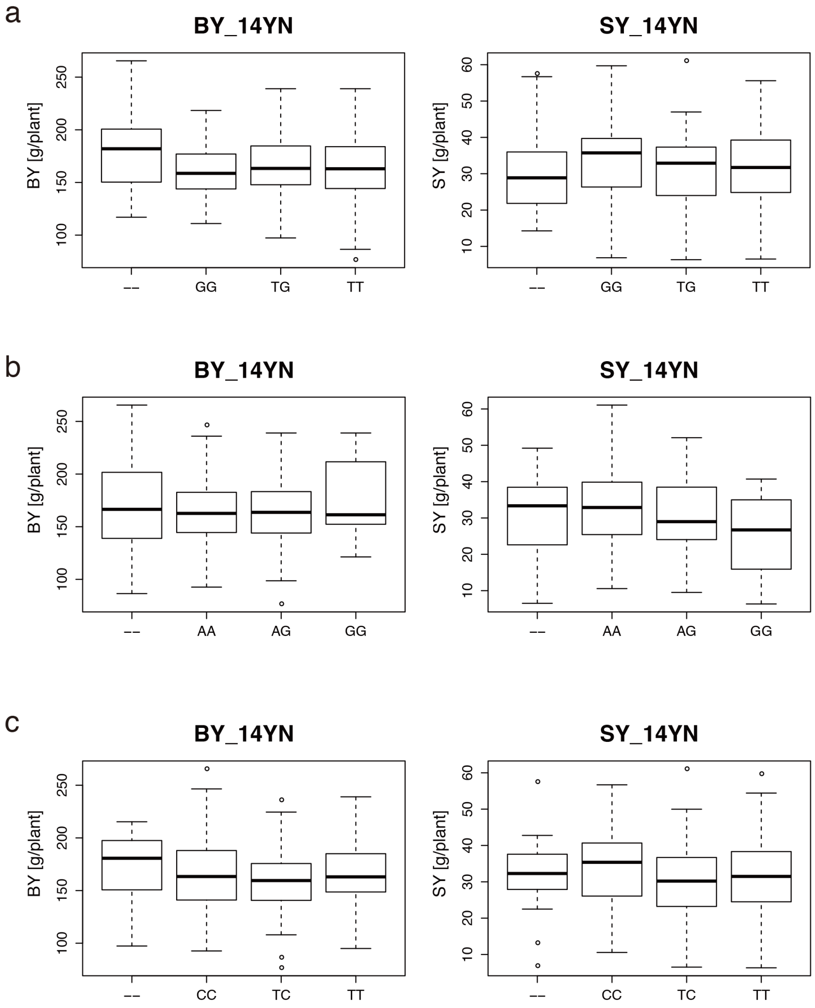

Supplementary Figure S5. Boxplot representing the phenotypic differences for BY and SY between lines carrying different alleles at high yield region YN. (a) Phenotypic difference between lines carrying different alleles, GG, TG and TT, of locus Bn_scaff_19170_1_p1132242. (b) Phenotypic difference between lines carrying different alleles, AA, AG and GG, of locus Bn_scaff_19526_1_p67 (c) Phenotypic difference between lines carrying different alleles, CC, TC and TT, of locus Bn_A09_p25738298.

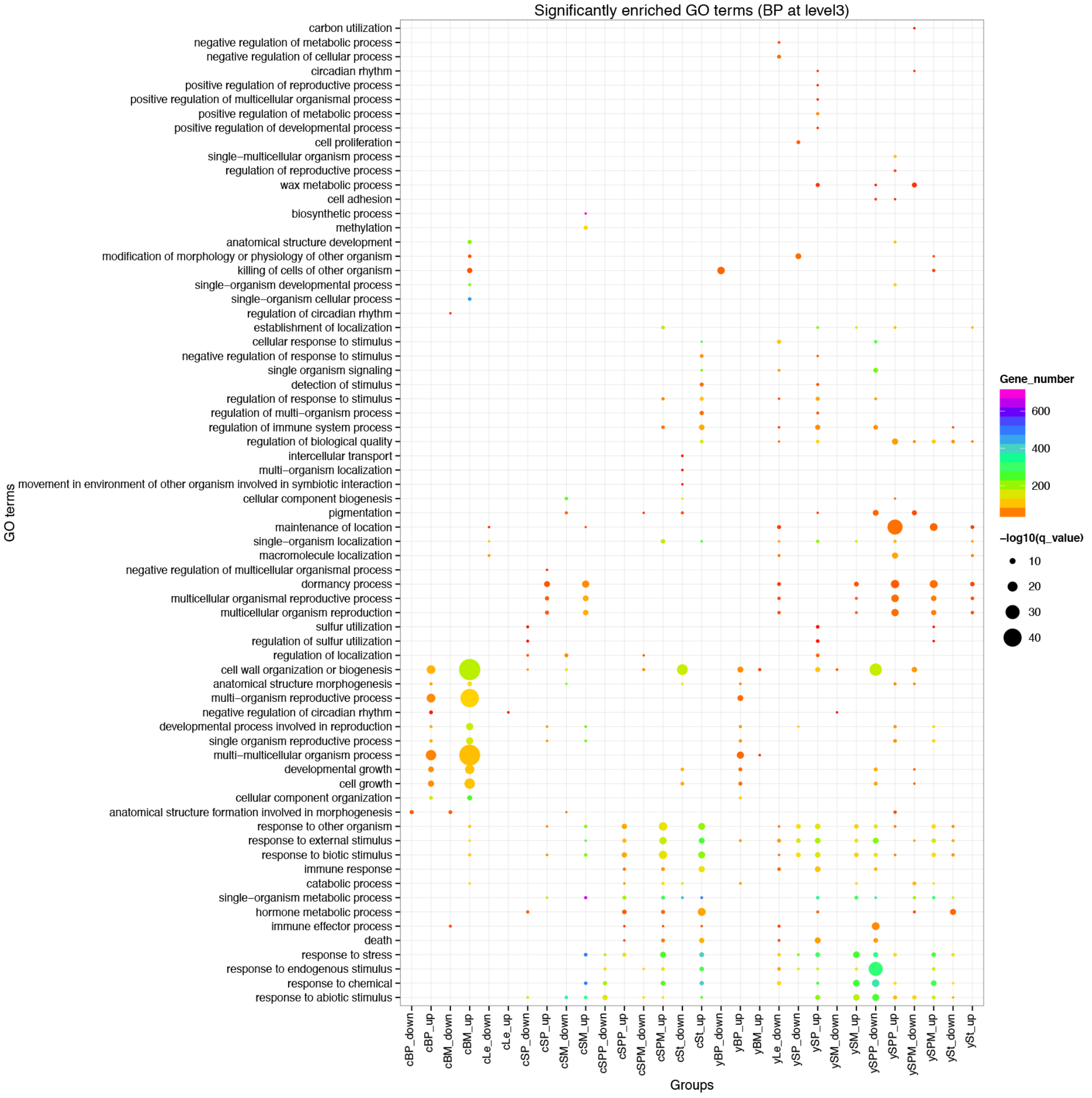

Supplementary Figure S5. Significantly overrepresented GO Biological Process terms (level 3) of DEGs. First letters (c and y) represent cultivation regions CQ and YN, respectively. SPP: silique pericarps on the primary branch, BP: buds on the primary branch, SP: seeds harvested 20 days after flowering on the primary branch, Le: leaves, St: stems, SPM: silique pericarps on the main inflorescence, BM: buds on the main inflorescence, SM: seeds harvested 20 days after flowering on the main inflorescence. The "up" and "down" indicate that the GO enrichment results were derived from up- and down-regulated genes, respectively.

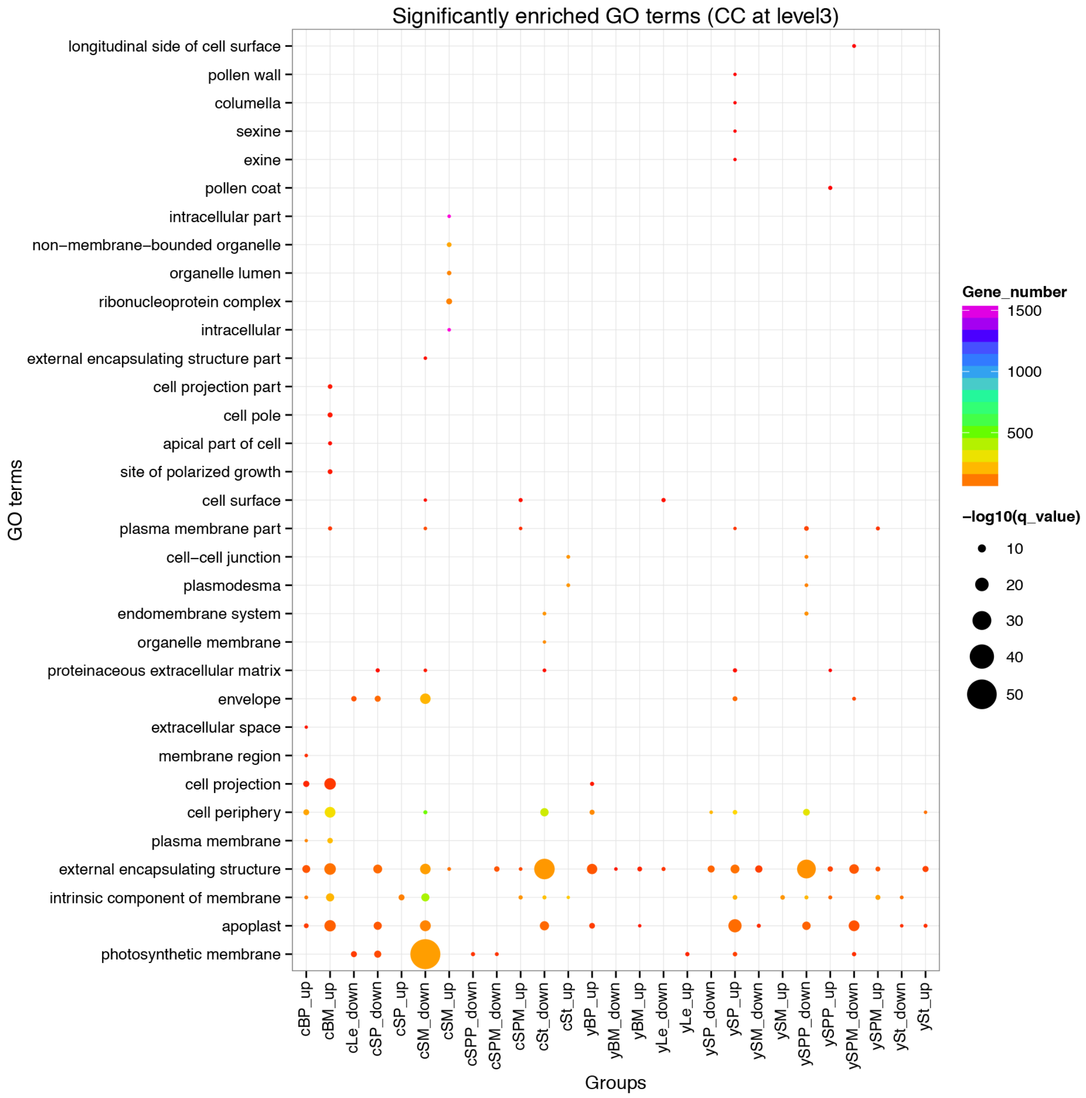

Supplementary Figure S6. Significantly overrepresented GO Cell Component terms (level 3) of DEGs. Abbreviations of sample names are the same as in Supplementary Figure S3.

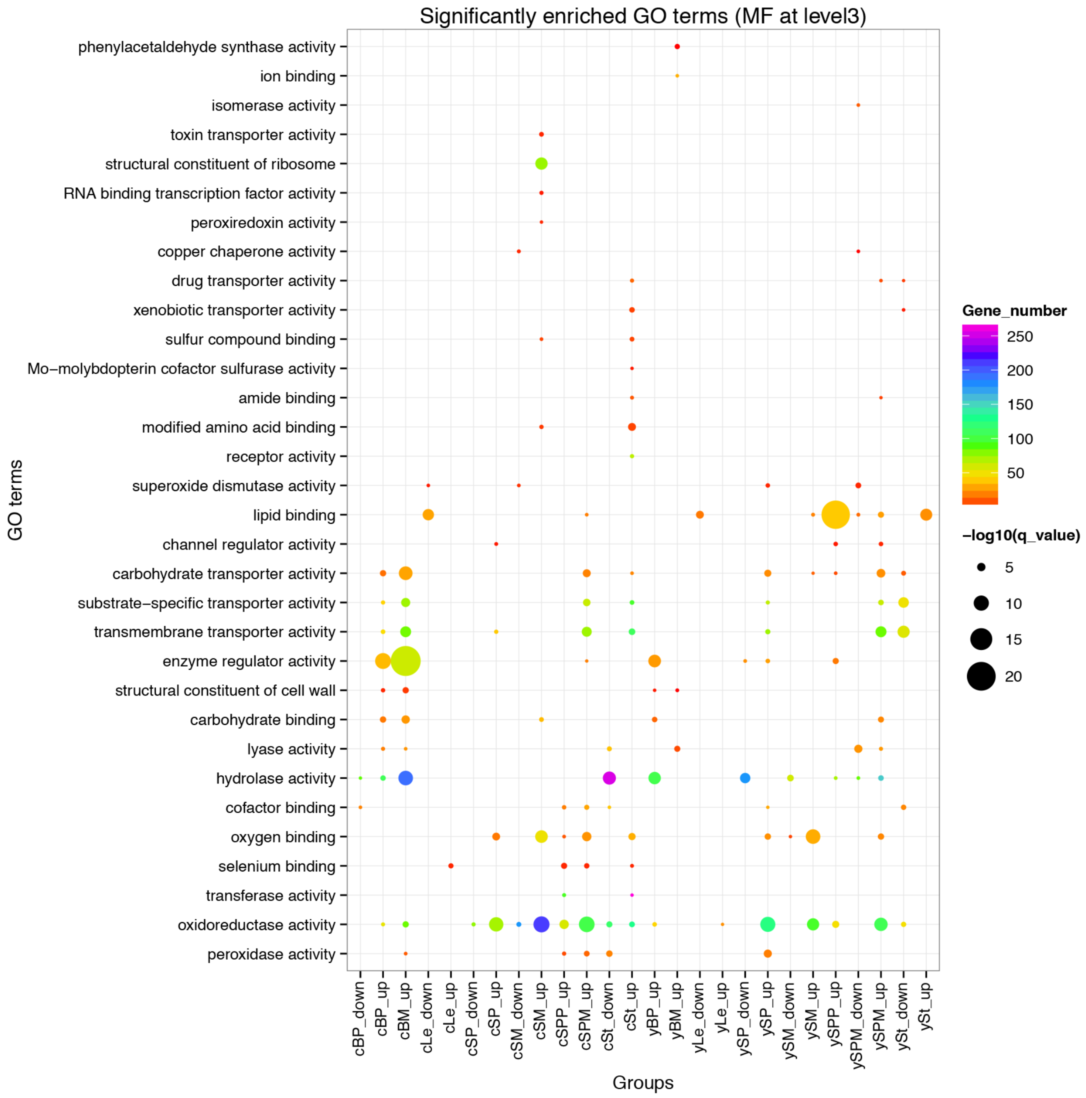

Supplementary Figure S7. Significantly overrepresented GO Molecular Function terms (level 3) of DEGs. Abbreviations of sample names are the same as in Supplementary Figure S3.

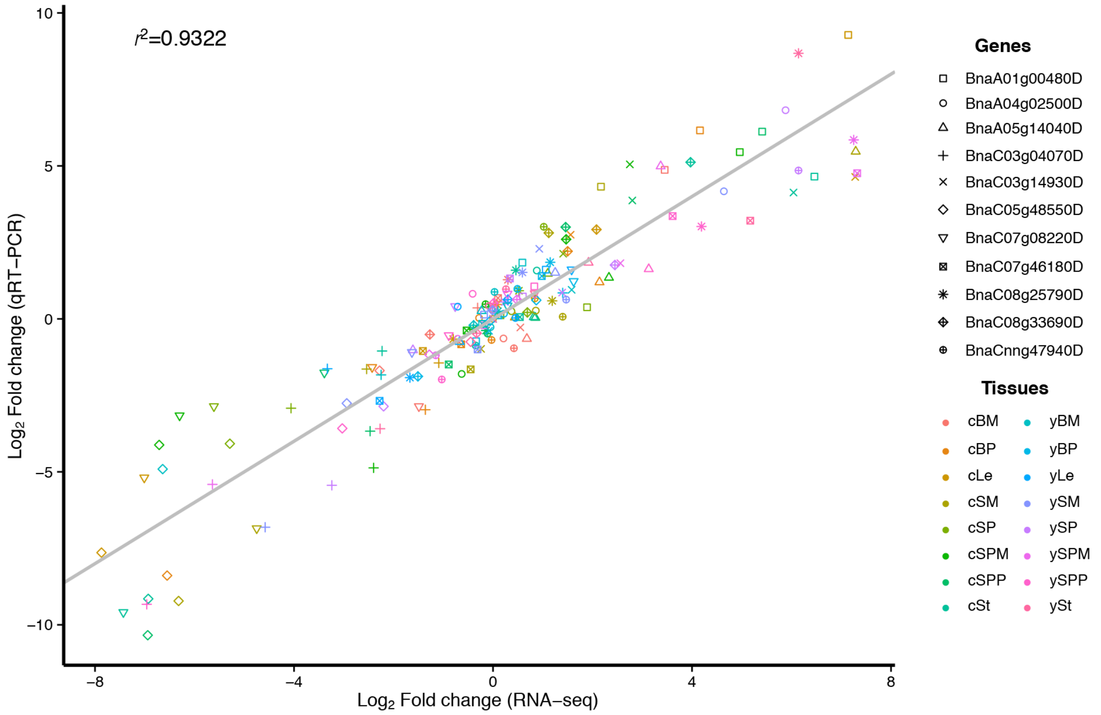

Supplementary Figure S8. Comparison of gene expression values obtained by RNA-seq and qRT-PCR. Fold changes were calculated for 10 DEGs in 8 tissues from plants grown at both CQ and YN, and a high correlation (r^2 = 0.9322) was observed between the results derived from the two techniques.

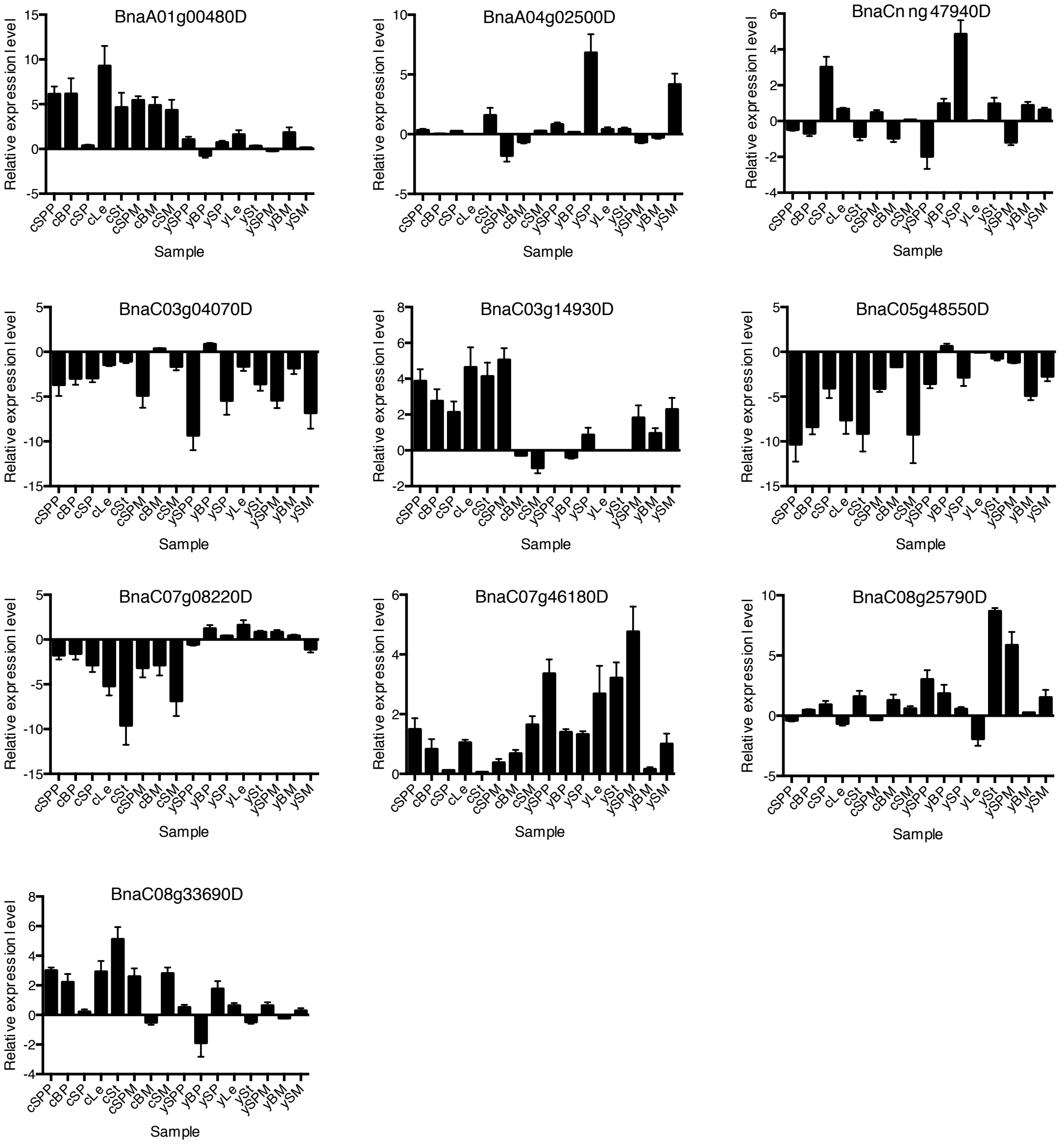

Supplementary Figure S9. qRT-PCR verification of DEGs. First letters (c and y) represent cultivation regions CQ and YN, respectively. Values represent the average ± SD of two biological replicates with three technical replicates per sample.

Supplementary Table S1. Descriptions of *B. napus* accessions used in this study.

Accession number	Accession name	Country/Region	Type	Sub-population [a]
B1	Ganyou5	Chongqing (China)	Winter	P1
B2	Nonglin42	Chongqing (China)	Winter	P1
B3	Kelina	Chongqing (China)	Winter	P1
B4	Huaiyou6	Chongqing (China)	Winter	P1
B5	Yan81-2	Chongqing (China)	Winter	P1
B6	28887	Chongqing (China)	Winter	P1
B7	Qianyou4	Chongqing (China)	Semi-winter	P1
B8	Qianyou331	Chongqing (China)	Semi-winter	P1
B9	Enyou73-1-2	Chongqing (China)	Winter	P1
B10	Niuerduo	Chongqing (China)	Winter	P1
B11	880101	Chongqing (China)	Winter	P1
B12	SWU40	Chongqing (China)	Winter	P1
B13	SWU42	Chongqing (China)	Winter	P1
B14	SWU43	Chongqing (China)	Winter	P1
B15	SWU44	Chongqing (China)	Winter	P1
B16	SWU45	Chongqing (China)	Winter	P1
B17	SWU46	Chongqing (China)	Winter	P1
B18	SWU47	Chongqing (China)	Winter	P1
B19	SWU48	Chongqing (China)	Winter	P1
B20	SWU52	Chongqing (China)	Winter	P1
B21	SWU53	Chongqing (China)	Winter	P1
B22	SWU56	Chongqing (China)	Winter	P1
B23	SWU59	Chongqing (China)	Winter	P1
B24	SWU65	Chongqing (China)	Winter	P1
B25	SWU82	Chongqing (China)	Winter	P1
B26	SWU83	Chongqing (China)	Winter	P1
B27	SWU92	Chongqing (China)	Winter	P1
B28	SWU101	Chongqing (China)	Winter	P1
B29	SWU106	Chongqing (China)	Winter	P1
B30	SWU108	Chongqing (China)	Winter	P1
B31	Chuanyou20	Sichuan (China)	Winter	P1
B32	Chuanyou18	Sichuan (China)	Winter	P1
B33	CY12NY-7	Sichuan (China)	Winter	P1
B34	CY12Q95406	Sichuan (China)	Winter	P1
B35	CY12Q8-7	Sichuan (China)	Winter	P1
B36	CY12QSZ06	Sichuan (China)	Winter	P1
B37	CY12QCWH-1	Sichuan (China)	Winter	P1
B38	CY12Q95108	Sichuan (China)	Winter	P1
B39	CY12Q21535-N3	Sichuan (China)	Winter	P1

B40	CY12PXW-4	Sichuan (China)	Winter	P1
B41	CY12PXW-6	Sichuan (China)	Winter	P1
B42	CY12PXW-9	Sichuan (China)	Winter	P1
B43	CY13PXW-17	Sichuan (China)	Winter	P1
B44	CY14PXW-18	Sichuan (China)	Winter	P1
B45	CY15PXW-31	Sichuan (China)	Winter	P1
B46	CY16PXW-35	Sichuan (China)	Winter	P1
B47	CY17PXW-58	Sichuan (China)	Winter	P1
B48	CY18PXW-62	Sichuan (China)	Winter	P1
B49	CY19PXW-65	Sichuan (China)	Winter	P1
B50	CY20PXW-66	Sichuan (China)	Winter	P1
B51	CY21PXW-84	Sichuan (China)	Winter	P1
B52	CY12GJ-1	Sichuan (China)	Winter	P1
B53	wx1025	Hunan (China)	Winter	P1
B54	wx10213	Hunan (China)	Winter	P1
B55	wx10296	Hunan (China)	Winter	P1
B56	wx10315	Hunan (China)	Winter	P1
B57	10-1043	Hunan (China)	Winter	P1
B58	10-1047	Hunan (China)	Winter	P1
B59	10-1061	Hunan (China)	Winter	P1
B60	10-1070	Hunan (China)	Winter	P1
B61	10-804	Hunan (China)	Winter	P1
B62	10-1358	Hunan (China)	Winter	P1
B63	1472	Hunan (China)	Winter	P1
B64	Xiangyou13	Hunan (China)	Winter	P1
B65	Xiangyou15	Hunan (China)	Winter	P1
B66	Xiangyou11	Hunan (China)	Winter	P1
B67	740	Hunan (China)	Winter	P1
B68	631	Hunan (China)	Winter	P1
B69	613	Hunan (China)	Winter	P1
B70	783	Hunan (China)	Winter	P1
B71	782	Hunan (China)	Winter	P1
B72	YB3	Hunan (China)	Winter	P1
B73	1360	Hunan (China)	Winter	P1
B74	563	Hunan (China)	Winter	P1
B75	WX10329	Hunan (China)	Semi-winter	P1
B76	santana	Hunan (China)	Winter	P1
B77	1281	Hunan (China)	Semi-winter	P1
B78	509	Hunan (China)	Semi-winter	P1
B79	1368	Hunan (China)	Semi-winter	P1
B80	1322	Hunan (China)	Semi-winter	P1
B81	1252	Hunan (China)	Semi-winter	P1

B82	1321	Hunan (China)	Semi-winter	P1
B83	07022	Hubei (China)	Semi-winter	P1
B84	07094	Hubei (China)	Semi-winter	P1
B85	07016	Hubei (China)	Semi-winter	P1
B86	9F087	Hubei (China)	Semi-winter	P1
B87	97096	Hubei (China)	Semi-winter	P1
B88	97097	Hubei (China)	Semi-winter	P1
B89	07189	Hubei (China)	Semi-winter	P1
B90	07191	Hubei (China)	Semi-winter	P1
B91	07037	Hubei (China)	Semi-winter	P1
B92	RQ011	Hubei (China)	Spring	P1
B93	RR009	Hubei (China)	Spring	P1
B94	RR002	Hubei (China)	Spring	P1
B95	97177	Hubei (China)	Semi-winter	P1
B96	96021	Hubei (China)	Semi-winter	P2
B97	96063	Hubei (China)	Semi-winter	P1
B98	01111	Hubei (China)	Semi-winter	P1
B99	01570	Hubei (China)	Semi-winter	P1
B100	9Bao22	Hubei (China)	Semi-winter	P1
B101	01188	Hubei (China)	Semi-winter	P1
B102	02354	Hubei (China)	Semi-winter	P1
B103	02359	Hubei (China)	Semi-winter	P1
B104	02365	Hubei (China)	Semi-winter	P1
B105	93205	Hubei (China)	Semi-winter	P1
B106	93210	Hubei (China)	Semi-winter	P1
B107	Nca	Hubei (China)	Semi-winter	P1
B108	Zhongshuang4	Hubei (China)	Semi-winter	P1
B109	Zhongshuang9	Hubei (China)	Semi-winter	P1
B110	Zhongshuang11	Hubei (China)	Semi-winter	P1
B111	2011-6200	Hubei (China)	Semi-winter	P1
B112	2011-6308	Hubei (China)	Semi-winter	P2
B113	2011-7103	Hubei (China)	Semi-winter	P1
B114	2012-11526	Hubei (China)	Semi-winter	P1
B115	2012-3448	Hubei (China)	Semi-winter	P2
B116	2012-3546	Hubei (China)	Semi-winter	P1
B117	2012-4531	Hubei (China)	Semi-winter	P1
B118	2012-5086	Hubei (China)	Semi-winter	P1
B119	2012-5113	Hubei (China)	Semi-winter	P1
B120	2012-8327	Hubei (China)	Semi-winter	P1
B121	2012-8355	Hubei (China)	Semi-winter	P1
B122	2012-8380	Hubei (China)	Semi-winter	P1
B123	2012-8998	Hubei (China)	Semi-winter	P1

B124	2012-9323	Hubei (China)	Semi-winter	P1
B125	2012-9354	Hubei (China)	Semi-winter	P1
B126	2012-9380	Hubei (China)	Semi-winter	P1
B127	2012-9478	Hubei (China)	Semi-winter	P1
B128	2012-9542	Hubei (China)	Semi-winter	P1
B129	2012-K8053	Hubei (China)	Semi-winter	P1
B130	R2	Hubei (China)	Semi-winter	P1
B131	Xiwang106	Hubei (China)	Semi-winter	P1
B132	Yangguang198	Hubei (China)	Semi-winter	P1
B133	Yangguang2009	Hubei (China)	Semi-winter	P1
B134	Zhongshuang10	Hubei (China)	Semi-winter	P1
B135	Zhongshuang12	Hubei (China)	Semi-winter	P1
B136	Zhongshuang4	Hubei (China)	Semi-winter	P1
B137	Zhongshuang6	Hubei (China)	Semi-winter	P1
B138	Zhongshuang7	Hubei (China)	Semi-winter	P1
B139	Zhongyou589	Hubei (China)	Semi-winter	P1
B140	Zhongyou821	Hubei (China)	Semi-winter	P1
B141	Huayou2	Hubei (China)	Semi-winter	P1
B142	Major	Hubei (China)	Winter	P1
B143	Huashuang2	Hubei (China)	Semi-winter	P1
B144	Aurora	Hubei (China)	Spring	P1
B145	Huayou13	Hubei (China)	Semi-winter	P1
B146	Rucabo	Hubei (China)	Spring	P2
B147	Huayou3	Hubei (China)	Semi-winter	P1
B148	Huayou14	Hubei (China)	Semi-winter	P1
B149	Ningyou1	Hubei (China)	Semi-winter	P1
B150	Ceres	Hubei (China)	Winter	P2
B151	11-9-700	Hubei (China)	Semi-winter	P1
B152	11-9-701	Hubei (China)	Semi-winter	P1
B153	11-9-702	Hubei (China)	Semi-winter	P1
B154	11-9-703	Hubei (China)	Semi-winter	P1
B155	11-9-704	Hubei (China)	Semi-winter	P1
B156	11-9-705	Hubei (China)	Semi-winter	P1
B157	11-9-706	Hubei (China)	Semi-winter	P1
B158	11-9-707	Hubei (China)	Semi-winter	P1
B159	11-P63-5	Hubei (China)	Semi-winter	P1
B160	11-P63-8	Hubei (China)	Semi-winter	P1
B161	11-P63-3	Hubei (China)	Semi-winter	P1
B162	11-P67	Hubei (China)	Semi-winter	P1
B163	09-P64-1	Hubei (China)	Semi-winter	P1
B164	10-Cong23	Hubei (China)	Semi-winter	P1
B165	10-Cong24	Hubei (China)	Semi-winter	P1

B166	10-Cong25	Hubei (China)	Semi-winter	P1
B167	10-Cong29	Hubei (China)	Semi-winter	P1
B168	10-Cong32	Hubei (China)	Semi-winter	P1
B169	10-Cong33	Hubei (China)	Semi-winter	P1
B170	10-Cong34	Hubei (China)	Semi-winter	P1
B171	10-Jiangpeng2	Hubei (China)	Semi-winter	P1
B172	10-Jiangpeng3	Hubei (China)	Semi-winter	P1
B173	11-7-103	Hubei (China)	Semi-winter	P1
B174	11-7-117	Hubei (China)	Semi-winter	P1
B175	11-7-125	Hubei (China)	Semi-winter	P1
B176	11-P74-8	Hubei (China)	Semi-winter	P1
B177	11-P74-13	Hubei (China)	Semi-winter	P1
B178	7-7766-74	Hubei (China)	Semi-winter	P1
B179	P18	Hubei (China)	Semi-winter	P1
B180	64Peng-10	Hubei (China)	Semi-winter	P1
B181	Shengguang77	Hubei (China)	Semi-winter	P1
B182	Jiayu17	Hubei (China)	Semi-winter	P1
B183	Jiayu25	Hubei (China)	Semi-winter	P1
B184	Jiayu16	Hubei (China)	Semi-winter	P1
B185	Jiayu31	Hubei (China)	Semi-winter	P1
B186	Huashuang5	Hubei (China)	Semi-winter	P1
B187	Huashuang4	Hubei (China)	Semi-winter	P1
B188	Jia972	Hubei (China)	Semi-winter	P1
B189	Huashuang128	Hubei (China)	Semi-winter	P1
B190	Jia904	Hubei (China)	Semi-winter	P1
B191	Jia908	Hubei (China)	Semi-winter	P1
B192	JiaPF190	Hubei (China)	Semi-winter	P1
B193	Jia915	Hubei (China)	Semi-winter	P1
B194	Jia922	Hubei (China)	Semi-winter	P1
B195	Jia951	Hubei (China)	Semi-winter	P1
B196	Jia917	Hubei (China)	Semi-winter	P1
B197	Jia923	Hubei (China)	Semi-winter	P1
B198	Jia931	Hubei (China)	Semi-winter	P1
B199	Jiayu05	Hubei (China)	Semi-winter	P1
B200	Jia963	Hubei (China)	Semi-winter	P2
B201	Huyou17	Shanghai (China)	Semi-winter	P1
B202	Huyou15	Shanghai (China)	Semi-winter	P1
B203	Huyou12	Shanghai (China)	Semi-winter	P2
B204	Ningyou18	Jiangsu (China)	Semi-winter	P1
B205	Ningyou16	Jiangsu (China)	Semi-winter	P1
B206	Ningyou14	Jiangsu (China)	Semi-winter	P1
B207	Ningyou12	Jiangsu (China)	Semi-winter	P1

B208	Shilijia	Jiangsu (China)	Semi-winter	P1
B209	Shilifeng	Jiangsu (China)	Semi-winter	P1
B210	Yangyou6	Lixia(Jiangsu, China)	Semi-winter	P1
B211	Yangyou5	Lixia(Jiangsu, China)	Semi-winter	P1
B212	Zhenyou3	Zhejiang(China)	Semi-winter	P1
B213	Hongyou3	Nanjiang(China)	Semi-winter	P1
B214	Suyou1	Jiangsu(China)	Semi-winter	P1
B215	Zheyou18	Zhejiang(China)	Semi-winter	P1
B216	Zheshuang72	Zhejiang(China)	Semi-winter	P1
B217	Zheshuang8	Zhejiang(China)	Semi-winter	P1
B218	Zheyou758	Zhejiang(China)	Semi-winter	P1
B219	Huyou14	Shanghai (China)	Semi-winter	P1
B220	Huyou18	Shanghai (China)	Semi-winter	P1
B221	Huyou19	Shanghai (China)	Semi-winter	P1
B222	Zheyou19	Zhejiang(China)	Semi-winter	P1
B223	Zheyou21	Zhejiang(China)	Semi-winter	P1
B224	Zheshuang6	Zhejiang(China)	Semi-winter	P1
B225	Wanyou15	Anhui(China)	Semi-winter	P1
B226	Wanyou16	Anhui(China)	Semi-winter	P1
B227	Wanyou20	Anhui(China)	Semi-winter	P1
B228	Wanyou29	Anhui(China)	Semi-winter	P1
B236	AGREV012	German	Spring	P2
B237	AGREV019	German	Spring	P2
B238	AGREV021	German	Spring	P2
B239	Topas	Sweden	Spring	P2
B240	cyclon	Denmark	Spring	P2
B241	Weijie	Canada	Spring	P2
B242	Sida	Canada	Spring	P2
B243	Zhizun	Canada	Spring	P1
B244	Haisheng	Canada	Spring	P2
B245	D2	Denmark	Spring	P2
B246	D3	Denmark	Spring	P2
B247	Qu	United States	Spring	P2
B248	11-985	Qinghai(China)	Spring	P2
B249	11-504	Qinghai(China)	Spring	P1
B250	11-997	Qinghai(China)	Spring	P2
B251	11-540	Qinghai(China)	Spring	P2
B252	11-1184	Qinghai(China)	Spring	P1
B253	10-758	Qinghai(China)	Spring	P2
B254	10-1230	Qinghai(China)	Spring	P2
B255	10-847	Qinghai(China)	Spring	P2
B256	11-1124	Qinghai(China)	Spring	P2

B257	Shan2A	Shanxi (China)	Seni-Winter	P1
B258	Shan2B	Shanxi (China)	Semi-winter	P1
B259	KenC1	Shanxi (China)	Semi-winter	P1
B260	P113	Shanxi (China)	Semi-winter	P1
B261	P158	Shanxi (China)	Semi-winter	P2
B262	P310	Shanxi (China)	Semi-winter	P1
B263	P312	Shanxi (China)	Semi-winter	P2
B264	P668	Shanxi (China)	Semi-winter	P1
B265	P685	Shanxi (China)	Semi-winter	P1
B266	A117	Shanxi (China)	Semi-winter	P1
B268	B250	Shanxi (China)	Semi-winter	P1
B269	B265	Shanxi (China)	Semi-winter	P1
B270	A109	Shanxi (China)	Semi-winter	P1
B271	B285	Shanxi (China)	Semi-winter	P2
B272	C052	Shanxi (China)	Semi-winter	P1
B273	GY270	Shanxi (China)	Semi-winter	P1
B274	GY282	Shanxi (China)	Semi-winter	P1
B275	GY284	Shanxi (China)	Semi-winter	P1
B276	B262	Henan(China)	Semi-winter	P1
B277	A82	Jiangxi(China)	Semi-winter	P1
B278	A98	Shanxi(China)	Semi-winter	P1
B279	B414	Xinjiang(China)	Semi-winter	P1
B280	B308	Sweden	Spring	P2
B281	A97	Sichuan(China)	Semi-winter	P2
B282	A148	Sweden	Spring	P1
B283	B431	Denmark	Spring	P2
B284	08-P35	Hubei (China)	Semi-winter	P1
B285	08-P36	Hubei (China)	Semi-winter	P2
B286	09-P32	Hubei (China)	Semi-winter	P2
B287	09-P36	Hubei (China)	Semi-winter	P2
B288	09-P37	Hubei (China)	Semi-winter	P1
B289	10-P10	Hubei (China)	Semi-winter	P1
B290	10-P29	Hubei (China)	Semi-winter	P2
B291	11-P30	Hubei (China)	Semi-winter	P1
B292	12-P24	Hubei (China)	Semi-winter	P2
B293	12-P25	Hubei (China)	Semi-winter	P1
B294	03II13	Gansu (China)	Spring	P2
B296	03I32B	Gansu (China)	Spring	P2
B297	964	Gansu (China)	Winter	P2
B298	DD1	Gansu (China)	Spring	P1
B299	gl302-1	Gansu (China)	Spring	P2
B300	06T9F	Gansu (China)	Spring	P2

B301	Longyou2	Gansu (China)	Spring	P2
B302	03II4B	Gansu (China)	Spring	P2
B303	03LF1	Gansu (China)	Spring	P2
B304	9852	Gansu (China)	Winter	P2
B305	9801C	Gansu (China)	Spring	P2
B306	986	Gansu (China)	Winter	P2
B307	876	Gansu (China)	Winter	P2
B308	Wu164	Gansu (China)	Spring	P2
B309	Longyou4	Gansu (China)	Spring	P2
B310	9889	Gansu (China)	Winter	P2
B311	06H7	Gansu (China)	Spring	P2
B312	Tianyou4	Gansu (China)	Winter	P2
B313	SWU01	Hubei (China)	Winter	P1
B314	SWU2	Chongqing (China)	Winter	P1
B315	IMC103	Chongqing (China)	Winter	P2
B316	SWU4	Chongqing (China)	Winter	P1
B317	Sophia	German	Spring	P2
B318	campina	German	Spring	P2
B319	Conny	German	Spring	P2
B320	Wesreo	German	Spring	P2
B321	Wase Chousen	German	Spring	P1
B322	Gogatsuna	German	Spring	P1
B323	Nakaee Chousen	German	Spring	P1
B324	Cat.No.117	German	Spring	P1
B325	90750	Chongqing (China)	Winter	P2
B326	Nonglin43	Chongqing (China)	Winter	P1
B327	SWU25	Tibet (China)	Winter	P1
B328	Youyan2	Chongqing (China)	Winter	P1
B329	SWU39	Sichuan (China)	Winter	P1
B330	SWU41	Chongqing (China)	Semi-winter	P1
B331	SWU49	Chongqing (China)	Semi-winter	P1
B332	SWU54	Chongqing (China)	Semi-winter	P1
B333	SWU57	Chongqing (China)	Semi-winter	P1
B334	SWU60	Chongqing (China)	Semi-winter	P1
B335	SWU61	Chongqing (China)	Semi-winter	P2
B336	SWU62	Chongqing (China)	Semi-winter	P2
B337	SWU63	Chongqing (China)	Semi-winter	P1
B338	SWU64	Chongqing (China)	Semi-winter	P1
B339	SWU66	Chongqing (China)	Semi-winter	P1
B340	SWU67	Chongqing (China)	Semi-winter	P1
B341	SWU68	Chongqing (China)	Semi-winter	P1
B342	SWU69	Chongqing (China)	Semi-winter	P1

B343	SWU70	Chongqing (China)	Semi-winter	P1
B344	SWU71	Chongqing (China)	Semi-winter	P1
B345	SWU74	Chongqing (China)	Semi-winter	P1
B346	SWU75	Chongqing (China)	Semi-winter	P1
B347	SWU76	Chongqing (China)	Semi-winter	P1
B348	SWU77	Chongqing (China)	Semi-winter	P1
B349	SWU80	Chongqing (China)	Semi-winter	P1
B350	SWU81	Chongqing (China)	Semi-winter	P1
B351	SWU84	Chongqing (China)	Semi-winter	P1
B352	SWU85	Chongqing (China)	Semi-winter	P1
B353	SWU97	Chongqing (China)	Semi-winter	P1
B354	SWU88	Chongqing (China)	Semi-winter	P1
B355	SWU89	Chongqing (China)	Semi-winter	P1
B356	SWU90	Chongqing (China)	Semi-winter	P1
B357	SWU93	Chongqing (China)	Semi-winter	P1
B358	SWU94	Chongqing (China)	Semi-winter	P1
B359	SWU95	Chongqing (China)	Semi-winter	P1
B360	SWU96	Chongqing (China)	Semi-winter	P1
B361	SWU99	Chongqing (China)	Semi-winter	P1
B362	SWU100	Chongqing (China)	Semi-winter	P1
B363	SWU102	Chongqing (China)	Semi-winter	P1
B364	SWU103	Chongqing (China)	Semi-winter	P1
B365	SWU104	Chongqing (China)	Semi-winter	P1
B366	SWU105	Chongqing (China)	Semi-winter	P1
B367	SWU107	Chongqing (China)	Semi-winter	P1
B368	SWU110	Chongqing (China)	Semi-winter	P1
B369	SWU111	Chongqing (China)	Semi-winter	P1
B370	SWU112	Chongqing (China)	Semi-winter	P1
B371	SWU113	Chongqing (China)	Semi-winter	P1
B372	SWU114	Chongqing (China)	Semi-winter	P1
B373	Zhongshuang10	Hubei (China)	Semi-winter	P1
B374	Zhongshuang6	Hubei (China)	Semi-winter	P1
B375	Zhongshuang7	Hubei (China)	Semi-winter	P1
B376	Zhongshuang12	Hubei (China)	Semi-winter	P1
B377	Zhongyou589	Hubei (China)	Semi-winter	P1
B378	Fuyou4	Hubei (China)	Spring	P1
B379	Zhen3736	Hubei (China)	Semi-winter	P1
B380	Zhen2609	Jiangsu(China)	Semi-winter	P1
B381	HX0352	Jiangsu (China)	Winter	P1
B383	Huyou21	Shanghai(China)	Semi-winter	P1
B384	Zheshuang3	Zhejiang(China)	Semi-winter	P1
B385	Zheyou21	Zhejiang(China)	Semi-winter	P1

B386	Wanyou20	Anhui(China)	Semi-winter	P1
B387	Wanyou12	Anhui(China)	Semi-winter	P1
B388	Wanyou7	Anhui(China)	Semi-winter	P1
B389	Hongyou3	Jiangsu(China)	Semi-winter	P1
B390	Zhenyou5	Hubei (China)	Semi-winter	P1
B391	Yangyou4	Hubei (China)	Semi-winter	P1
B392	Huyou15	Shanghai(China)	Semi-winter	P1
B393	Huyou16	Shanghai(China)	Semi-winter	P1
B394	Huyou17	Shanghai(China)	Semi-winter	P1
B395	Huyou18	Zhejiang(China)	Semi-winter	P1
B396	Zheshuang72	Zhejiang(China)	Semi-winter	P1
B397	Zheshuang8	Zhejiang(China)	Semi-winter	P1
B398	Zheyou50	Zhejiang(China)	Semi-winter	P1
B399	Suyou1	Jiangsu(China)	Semi-winter	P1
B400	Zhongshuang4	Hubei (China)	Semi-winter	P1
B401	Zhongshuang11	Hubei (China)	Semi-winter	P1
B402	Yangguang198	Hubei (China)	Semi-winter	P1
B403	Huahang901	Hubei (China)	Semi-winter	P1
B404	YangJ6711	Jiangsu (China)	Semi-winter	P1
B405	Yan6055	Jiangsu (China)	Semi-winter	P1
B406	Yangjian8	Jiangsu (China)	Semi-winter	P1
B407	Xiwang106	Hubei (China)	Semi-winter	P1
B408	Zheyou17	Zhejiang(China)	Semi-winter	P1
B409	Zhongshuang5	Hubei (China)	Semi-winter	P1
B410	Zhongyou821	Hubei (China)	Semi-winter	P1
B411	Qinyou1	Hubei (China)	Semi-winter	P1
B412	Weilong88	Hubei (China)	Semi-winter	P1
B413	Yanyou2	Hubei (China)	Semi-winter	P1
B414	Qinyou5	Hubei (China)	Semi-winter	P1
B415	Suyou4	Jiangsu (China)	Semi-winter	P1
B416	Fengyou9	Hubei (China)	Semi-winter	P1
B417	De68-12	Hubei (China)	Semi-winter	P1
B418	Monty	Hubei (China)	Spring_	P2
B419	Oscar	Hubei (China)	Semi-winter	P1
B420	Ningyou12	Hubei (China)	Semi-winter	P1
B421	Ningyou14	Hubei (China)	Semi-winter	P1
B422	Shilifeng	Hubei (China)	Semi-winter	P1
B423	Ningyou18	Hubei (China)	Semi-winter	P1
B424	Ningyou10	Hubei (China)	Semi-winter	P1
B425	Helios	Hubei (China)	Spring	P1
B426	Hector	Hubei (China)	Semi-winter	P1
B427	Mian96-203	Qinghai(China)	Spring	P1

B428	Qing662A	Hubei (China)	Semi-winter	P1
B429	699	Hubei (China)	Semi-winter	P1
B430	Canada2	Hubei (China)	Semi-winter	P1
B431	Zhongshuang2	Hubei (China)	Semi-winter	P1
B432	Zhongshuang9	Hubei (China)	Semi-winter	P1
B433	WH-12	Hubei (China)	Semi-winter	P1
B434	WH-15	Hubei (China)	Semi-winter	P2
B435	WH-17	Hubei (China)	Semi-winter	P1
B436	WH-19	Hubei (China)	Semi-winter	P1
B437	WH-20	Hubei (China)	Semi-winter	P1
B438	WH-23	Hubei (China)	Semi-winter	P1
B439	WH-24	Hubei (China)	Semi-winter	P1
B440	WH-25	Hubei (China)	Semi-winter	P1
B441	WH-26	Hubei (China)	Semi-winter	P1
B442	WH-27	Hubei (China)	Semi-winter	P1
B443	WH-28	Hubei (China)	Semi-winter	P1
B444	WH-29	Hubei (China)	Semi-winter	P1
B445	WH-30	Hubei (China)	Semi-winter	P1
B446	WH-31	Hubei (China)	Semi-winter	P1
B447	WH-33	Hubei (China)	Semi-winter	P1
B448	WH-37	Hubei (China)	Semi-winter	P2
B449	WH-38	Hubei (China)	Semi-winter	P2
B450	WH-41	Hubei (China)	Semi-winter	P2
B451	WH-42	Hubei (China)	Semi-winter	P2
B452	WH-43	Hubei (China)	Semi-winter	P2
B453	WH-45	Hubei (China)	Semi-winter	P2
B454	WH-49	Hubei (China)	Semi-winter	P1
B455	WH-50	Hubei (China)	Semi-winter	P2
B456	WH-55	Hubei (China)	Semi-winter	P2
B457	WH-56	Hubei (China)	Semi-winter	P2
B458	WH-57	Hubei (China)	Semi-winter	P1
B459	WH-58	Hubei (China)	Semi-winter	P1
B460	WH-59	Hubei (China)	Semi-winter	P1
B461	WH-60	Hubei (China)	Semi-winter	P2
B462	WH-61	Hubei (China)	Semi-winter	P2
B463	WH-62	Hubei (China)	Semi-winter	P2
B464	WH-63	Hubei (China)	Semi-winter	P1
B465	WH-81	Hubei (China)	Semi-winter	P1
B466	WH-83	Hubei (China)	Semi-winter	P1
B467	WH-85	Hubei (China)	Semi-winter	P1
B468	WH-88	Hubei (China)	Semi-winter	P1
B469	WH-93	Hubei (China)	Semi-winter	P2

B470	WH-95	Hubei (China)	Semi-winter	P2
B471	WH-100	Hubei (China)	Semi-winter	P2
B472	WH-127	Hubei (China)	Semi-winter	P2
B473	Yuyou1	Henan (China)	Semi-winter	P1
B474	COBRA	Hubei (China)	Winter	P2
B475	NY7	Hubei (China)	Semi-winter	P1
B476	Tapidor	Hubei (China)	Winter	P2
B477	Huayou6	Hubei (China)	Semi-winter	P1
B478	Huayou12	Hubei (China)	Semi-winter	P1
B479	Cubs root	Hubei (China)	Spring	P1
B480	Huayou10	Hubei (China)	Semi-winter	P1
B481	Bienvenu	Hubei (China)	Winter	P1
B482	Shengli	Hubei (China)	Semi-winter	P1
B483	ERAKE	Hubei (China)	Spring	P2
B484	Taisetsu	Hubei (China)	Vegetable	P1
B485	cresor	Hubei (China)	Spring	P2
B486	Daichousen	Hubei (China)	Semi-winter	P1
B487	comet	Hubei (China)	Spring	P2
B488	Niklas	Hubei (China)	Spring	P2
B489	Askari	Hubei (China)	Winter	P2
B490	chuosenshu	Hubei (China)	Vegetable	P1
B491	WESBROOK	Hubei (China)	Spring	P2
B492	Suigenshu	Hubei (China)	Spring	P1
B493	Huayou4	Hubei (China)	Semi-winter	P1
B494	Y1	Shanghai (China)	Semi-winter	P1
B495	Mijiaoduotou rapeseed	Shanghai (China)	Semi-winter	P1
B496	Aijishengli	Shanghai (China)	Semi-winter	P1
B497	Caojingshengli	Shanghai (China)	Semi-winter	P1
B498	Huyou 3	Shanghai (China)	Semi-winter	P1
B499	Shaoyeqing	Shanghai (China)	Semi-winter	P1
B500	African rapeseed ruhuanghua	Shanghai (China)	Semi-winter	P1
B501	Hujizao	Shanghai (China)	Semi-winter	P1
B502	Caoyou 2	Shanghai (China)	Semi-winter	P1
B503	Huyou 16	Shanghai (China)	Semi-winter	P1
B504	Fengding 240	Jiangsu (China)	Semi-winter	P1
B505	Quanzi rapeseed	Jiangsu (China)	Semi-winter	P2
B506	Dahuaqiu	Jiangsu (China)	Semi-winter	P1
B507	Rongxuan	Jiangsu (China)	Semi-winter	P1
B508	Ningyou 10	Jiangsu (China)	Semi-winter	P1
B509	Ningyou 8	Jiangsu (China)	Semi-winter	P1
B510	Ningyou 6	Jiangsu (China)	Semi-winter	P1

B511	Duoyou 1	Jiangsu (China)	Semi-winter	P1
B512	Huaiyou 6	Jiangsu (China)	Semi-winter	P1
B513	Huaiyou 12	Jiangsu (China)	Semi-winter	P1
B514	Peixuan 170	Jiangsu (China)	Semi-winter	P1
B515	Guangde 138	Anhui (China)	Semi-winter	P1
B516	Guangde 8104	Anhui (China)	Semi-winter	P1
B517	Dangyouzao 1	Anhui (China)	Semi-winter	P1
B518	Guangde 761	Anhui (China)	Semi-winter	P1
B519	Tonglinghuaye	Anhui (China)	Semi-winter	P1
B520	Chu 610	Anhui (China)	Semi-winter	P1
B521	Chu 107	Anhui (China)	Semi-winter	P1
B522	Su 84-6	Anhui (China)	Semi-winter	P1
B523	Huanyouzao	Anhui (China)	Semi-winter	P1
B524	Chuyou1	Anhui (China)	Semi-winter	P1
B525	Chuxianbaihua	Anhui (China)	Semi-winter	P1
B526	Jie 65-1	Zhejiang (China)	Semi-winter	P1
B527	Shenhuang1	Shanghai (China)	Semi-winter	P1
B528	Zheyou 601	Zhejiang (China)	Semi-winter	P1
B529	Sangao rapeseed	Zhejiang (China)	Semi-winter	P1
B530	Zaofeng 1	Zhejiang (China)	Semi-winter	P1

[a] Population was assigned based on STRUCTURE analysis results in our previous study (Qu et al., 2015).

Supplementary Table S2. List of markers associated with HI and four HI-related traits.

Trait	Env.	SNP marker	Chr.	Position	*P*-Value	R^2	Favorable allele	Unfavorable allele
BY	13CQ	Bn-A03-p2948394	A03	2510281	1.68E-05	0.0544	AA	GG
BY	13CQ	Bn-A03-p4256940	A03	3794804	2.41E-05	0.0527	GG	AA
BY	13CQ	Bn-A03-p7643795	A03	6944642	2.16E-05	0.0533		
BY	13CQ	Bn-A03-p8475614	A03	7780068	8.75E-06	0.0575	TT	GG
BY	13CQ	Bn-A03-p9129107	A03	8414874	2.13E-05	0.0533	AA	GG
BY	13CQ	Bn-A03-p15675871	A03	14735140	1.86E-05	0.054		
BY	13CQ	Bn-A03-p18873704	A03	17888586	1.92E-05	0.0538	GG	AA
BY	14CQ	Bn-A03-p19751864	A03	18716830	1.27E-05	0.0347		
BY	13CQ	Bn-A03-p20375514	A03	19247585	3.47E-08	0.0836	AA	GG
BY	13CQ	Bn-A03-p21904842	A03	20689460	1.13E-05	0.0563	AA	GG
BY	13CQ	Bn-A04-p2222852	A04	1752487	2.39E-05	0.0528	AA	GG
BY	13CQ	Bn-A04-p8117244	A04	9402482	1.42E-05	0.0552	AA	GG
BY	13CQ	Bn-A04-p12759499	A04	13429641	1.00E-05	0.0568	CC	TT
BY	13CQ	Bn-A04-p15108913	A04	15545719	3.30E-06	0.0621	AA	GG
BY	13CQ	Bn-A04-p17736387	A04	17846200	1.90E-05	0.0539	AA	GG
BY	13CQ	Bn-A05-p822597	A05	958179	9.52E-06	0.0571	AA	TT
BY	13CQ	Bn-A05-p6542040	A05	6205280	2.82E-05	0.052	GG	AA
BY	13CQ	Bn-A06-p11919396	A06	14582267	3.39E-06	0.0619	CC	TT
BY	13CQ	Bn-A06-p14576278	A06	16129554	4.66E-06	0.0604	TT	GG
BY	13CQ	Bn-A06-p16406113	A06	17919297	6.12E-06	0.0592	GG	TT
BY	13CQ	Bn-A06-p16994210	A06	18408096	9.06E-07	0.0681	TT	CC
BY	13CQ	Bn-A06-p18065846	A06	19443728	1.58E-05	0.0547	TT	CC
BY	13CQ	Bn-A06-p24501540	A06	23444533	8.35E-07	0.0685		
BY	13CQ	Bn-scaff_17708_1-p169681	A07	1250011	5.03E-07	0.0709	AA	TT
BY	13CQ	Bn-A07-p12412116	A07	14546056	3.11E-05	0.0515	TT	GG
BY	13CQ	Bn-A07-p12878986	A07	14975988	1.29E-05	0.0557	TT	CC
BY	13CQ	Bn-scaff_15818_1-p2633791	A07	15988008	4.27E-06	0.0609	GG	TT
BY	13CQ	Bn-A07-p14594344	A07	16471338	6.76E-06	0.0587	CC	AA
BY	13CQ	Bn-A07-p14958606	A07	16830449	5.46E-06	0.0597	GG	AA
BY	13CQ	Bn-A07-p22034462	A07	23418663	1.09E-05	0.0564	GG	TT
BY	13CQ	Bn-A08-p1068698	A08	984273	7.03E-06	0.0585	AA	GG
BY	13CQ	Bn-A08-p1089123	A08	1000449	2.70E-05	0.0522	CC	TT
BY	13CQ	Bn-A08-p3444437	A08	2856532	8.76E-07	0.0683	AA	GG
BY	13CQ	Bn-A08-p4293226	A08	3694293	1.69E-05	0.0544	TT	CC
BY	13CQ	Bn-A08-p7814328	A08	6786884	1.74E-06	0.0651	AA	GG
BY	13CQ	Bn-A08-p8543675	A08	7389541	5.94E-07	0.0701	CC	TT
BY	13CQ	Bn-scaff_27039_1-p197834	A08	7397046	3.84E-06	0.0613	CC	TT
BY	13CQ	Bn-A08-p10795996	A08	8702202	2.79E-05	0.0521	AA	GG

BY	13CQ	Bn-A08-p11329864	A08	9184409	2.19E-05	0.0532	TT	CC
BY	13CQ	Bn-A08-p12556455	A08	10335312	2.67E-05	0.0523	CC	TT
BY	13CQ	Bn-A08-p12752628	A08	10449877	4.00E-08	0.0829	AA	GG
BY	13CQ	Bn-A08-p13376746	A08	11121237	7.87E-06	0.058	AA	GG
BY	13CQ	Bn-A08-p17108442	A08	14587172	2.71E-05	0.0522	CC	TT
BY	13CQ	Bn-A08-p17193797	A08	14679373	8.68E-07	0.0615		
BY	13CQ	Bn-A08-p19676653	A08	17007108	1.06E-05	0.0566	CC	TT
BY	13CQ	Bn-A08-p20354609	A08	17822431	9.49E-07	0.0679	CC	TT
BY	13CQ	Bn-A09-p10306216	A09	9527218	4.27E-06	0.0608	CC	TT
BY	13CQ	Bn-A09-p21224256	A09	18562826	1.32E-06	0.0664	TT	CC
BY	13CQ	Bn-A07-p9535820	A09	19578645	1.64E-05	0.0545	CC	AA
BY	13CQ	Bn-A09-p33694154	A09	30983144	1.35E-06	0.0662	CC	TT
BY	14CQ	Bn-A09-p35604358	A09	32733944	2.55E-05	0.0438	GG	AA
BY	13CQ	Bn-A10-p7410271	A10	9082874	1.40E-05	0.0553	TT	CC
BY	13CQ	Bn-scaff_17827_1-p271972	C01	7529799	2.66E-06	0.0631	GG	AA
BY	13CQ	Bn-scaff_22790_1-p370880	C01	30305788	1.15E-05	0.0562	TT	CC
BY	13CQ	Bn-C13594816-p86	C02	40111275	3.34E-06	0.062	AA	GG
BY	14CQ	Bn-scaff_17109_4-p95949	C02	40759539	2.69E-05	0.0384		
BY	14CQ	Bn-scaff_17109_1-p1144887	C02	41209839	2.47E-05	0.0439	TT	CC
BY	13CQ	Bn-scaff_17623_1-p1013272	C02	42483650	1.14E-05	0.0563	CC	TT
BY	13CQ	Bn-scaff_22728_1-p360792	C03	6151071	6.48E-06	0.0589	TT	CC
BY	13CQ	Bn-scaff_18322_1-p918195	C03	8116755	8.09E-07	0.0687	AA	TT
BY	13CQ	Bn-scaff_16002_1-p1569056	C03	12839150	4.27E-06	0.0608	GG	TT
BY	13CQ	Bn-scaff_18482_1-p437945	C03	19927672	2.92E-06	0.0626	TT	CC
BY	13CQ	Bn-scaff_17521_1-p807610	C03	21410571	1.66E-06	0.0653	TT	CC
BY	13CQ	Bn-scaff_17440_1-p761980	C03	47656790	1.38E-05	0.0554	GG	AA
BY	13CQ	Bn-scaff_16182_1-p319123	C03	51969206	8.87E-06	0.0574	CC	TT
BY	13CQ	Bn-scaff_15703_1-p53309	C03	52357539	4.30E-06	0.0608	CC	AA
BY	13CQ	Bn-A08-p293480	C03	60268012	4.28E-06	0.0608	AA	GG
BY	13CQ	Bn-scaff_18712_1-p602624	C04	14356066	2.67E-06	0.063	GG	TT
BY	13CQ	Bn-scaff_19049_1-p163830	C04	27480077	1.11E-06	0.0603	CC	AA
BY	13CQ	Bn-A04-p10903353	C04	37955630	4.92E-06	0.0602	CC	AA
BY	13CQ	Bn-A04-p15456188	C04	44583867	2.97E-05	0.0456		
BY	13CQ	Bn-scaff_21884_1-p235276	C05	4305731	1.31E-05	0.0556	GG	AA
BY	14CQ	Bn-scaff_18181_1-p620712	C05	7041784	1.72E-05	0.0453	TT	GG
BY	13CQ	Bn-scaff_18181_1-p573554	C05	7093460	1.99E-05	0.0536	AA	GG
BY	13CQ	Bn-scaff_16770_1-p684639	C05	35242768	7.05E-07	0.0624		
BY	13CQ	Bn-scaff_22310_1-p243575	C07	7893119	8.30E-06	0.0577	GG	TT

BY	13CQ	Bn-scaff_18181_1-p1849246	C07	34322840	6.29E-08	0.0807	CC	TT
BY	13CQ	Bn-scaff_16110_1-p1990416	C07	42889038	8.27E-06	0.0578	GG	AA
BY	13CQ	Bn-scaff_23765_1-p208	C08	7612606	1.18E-05	0.0561	AA	CC
BY	13CQ	Bn-scaff_16766_1-p179061	C08	9447598	1.14E-05	0.0499		
BY	13CQ	Bn-scaff_18602_1-p278628	C08	16438368	3.50E-07	0.0726	CC	AA
BY	13CQ	Bn-scaff_21786_1-p111311	C08	19631438	6.29E-06	0.059	CC	AA
BY	13CQ	Bn-scaff_16197_1-p1970599	C08	32209089	4.23E-07	0.0717	CC	TT
BY	13CQ	Bn-scaff_16197_1-p1087526	C08	33007840	1.22E-06	0.0667	GG	TT
BY	13CQ	Bn-scaff_16445_1-p968765	C08	35812498	3.32E-06	0.062	GG	AA
BY	13CQ	Bn-scaff_18100_1-p119095	C09	13746357	1.70E-05	0.0544	CC	TT
BY	13CQ	Bn-scaff_17088_1-p327069	C09	40117533	1.15E-05	0.0562	AA	GG
BY	13CQ	Bn-scaff_22082_1-p272234	C09	44582838	4.89E-06	0.0602	TT	CC
CBY	14CQ	Bn-A03-p19751864	A03	18716830	2.61E-05	0.0371		
CBY	13CQ	Bn-A03-p20375514	A03	19247585	2.78E-06	0.0532	AA	GG
CBY	14CQ	Bn-A06-p4436168	A06	4266288	1.64E-05	0.0524	AA	CC
CBY	13CQ	Bn-A06-p24501540	A06	23444533	5.76E-06	0.0503		
CBY	13CQ	Bn-scaff_17708_1-p169681	A07	1250011	4.01E-06	0.0517	AA	TT
CBY	13CQ	Bn-A07-p12878986	A07	14975988	2.58E-05	0.0443	TT	CC
CBY	13CQ	Bn-A07-p14594344	A07	16471338	1.80E-05	0.0458	CC	AA
CBY	13CQ	Bn-A07-p22034462	A07	23418663	1.68E-05	0.0461	GG	TT
CBY	13CQ	Bn-A08-p3444437	A08	2856532	8.00E-07	0.0581	AA	GG
CBY	13CQ	Bn-A08-p7814328	A08	6786884	2.40E-05	0.0446	AA	GG
CBY	13CQ	Bn-A08-p8543675	A08	7389541	7.60E-06	0.0492	CC	TT
CBY	13CQ	Bn-A08-p12752628	A08	10449877	5.52E-07	0.0596	AA	GG
CBY	13CQ	Bn-A08-p17108442	A08	14587172	1.93E-05	0.0455	CC	TT
CBY	14VTL	Bn-A08-p20314411	A08	17783149	2.43E-05	0.0809	AA	GG
CBY	13CQ	Bn-A08-p20354609	A08	17822431	5.04E-06	0.0508	CC	TT
CBY	13CQ	Bn-A09-p33694154	A09	30983144	1.02E-05	0.048	CC	TT
CBY	14YN-CQ	Bn-scaff_22563_1-p140785	C01	21627170	3.11E-05	0.0824	AA	GG
CBY	13CQ	Bn-scaff_18482_1-p437945	C03	19927672	2.14E-05	0.0451	TT	CC
CBY	13CQ	Bn-scaff_17521_1-p807610	C03	21410571	2.67E-05	0.0442	TT	CC
CBY	13CQ	Bn-A08-p293480	C03	60268012	2.99E-05	0.0438	AA	GG
CBY	13CQ	Bn-scaff_16770_1-p684639	C05	35242768	6.59E-06	0.0443		
CBY	13CQ	Bn-scaff_18181_1-p1849246	C07	34322840	1.53E-06	0.0555	CC	TT
CBY	13CQ	Bn-scaff_23765_1-p208	C08	7612606	3.04E-05	0.0437	AA	CC
CBY	13CQ	Bn-scaff_18602_1-p278628	C08	16438368	7.06E-06	0.0495	CC	AA

CBY	13CQ	Bn-scaff_16197_1-p1970599	C08	32209089	6.28E-06	0.0499	CC	TT
CBY	13CQ	Bn-scaff_16197_1-p1087526	C08	33007840	4.49E-06	0.0513	GG	TT
HI	14YN-CQ	Bn-A01-p5601232	A01	5121857	8.83E-06	0.0857	AA	CC
HI	13CQ	Bn-A01-p23281709	A01	19147254	2.03E-05	0.0478	GG	AA
HI	13CQ	Bn-A02-p10067386	A02	6895619	5.37E-06	0.0534	CC	TT
HI	14VTL	Bn-A06-p23865356	A06	22856804	5.55E-06	0.0868	CC	TT
HI	14YN-CQ	Bn-A06-p23865356	A06	22856804	2.43E-06	0.0961	CC	TT
HI	14YN-CQ	Bn-A06-p23873505	A06	22863780	6.13E-06	0.0867	AA	GG
HI	13CQ	Bn-A02-p337457	A07	12971098	9.62E-06	0.0509	TT	CC
HI	14VTL	Bn-scaff_15763_1-p506301	A07	15040520	2.98E-05	0.0661		
HI	14YN-CQ	Bn-scaff_15763_1-p506301	A07	15040520	1.63E-05	0.0743		
HI	14VTL	Bn-A07-p13942362	A07	15872128	2.92E-05	0.0551		
HI	13CQ	Bn-A07-p14969298	A07	16848134	5.36E-06	0.0534	AA	GG
HI	13CQ	Bn-A07-p16030550	A07	17932324	2.55E-05	0.0468	TT	CC
HI	14CQ	Bn-A07-p16721483	A07	18673520	1.06E-05	0.0476	AA	GG
HI	13CQ	Bn-A09-p1680594	A09	2250183	6.56E-06	0.0525	TT	GG
HI	13CQ	Bn-A10-p14384277	A10	14329378	2.03E-05	0.0478	CC	TT
HI	13CQ	Bn-A10-p15021776	A10	14967127	5.23E-06	0.0535	CC	TT
HI	13CQ	Bn-A10-p15022346	A10	14967696	6.02E-06	0.0529	CC	TT
HI	14YN	Bn-A10-p15287710	A10	16201895	2.49E-05	0.0786	TT	GG
HI	14VTL	Bn-scaff_24236_1-p370074	C01	20652274	2.57E-05	0.0762	TT	AA
HI	14VTL	Bn-scaff_19183_1-p243628	C01	21070857	2.32E-05	0.0677		
HI	14VTL	Bn-scaff_19183_1-p184833	C01	21120075	2.58E-05	0.0761		
HI	14VTL	Bn-scaff_16691_1-p967276	C01	21763061	3.13E-05	0.0748	CC	TT
HI	14YN-CQ	Bn-scaff_20646_1-p915635	C03	17143748	3.24E-05	0.0812	AA	GG
HI	14YN-CQ	Bn-scaff_20646_1-p144289	C03	17685634	2.16E-05	0.0825	GG	TT
HI	13CQ	Bn-scaff_16876_1-p662487	C04	34220432	2.90E-05	0.0463	GG	AA
HI	13CQ	Bn-scaff_20125_1-p522233	C05	10643948	2.48E-06	0.0566	CC	TT
HI	14VTL	Bn-scaff_15763_1-p506136	C06	20078539	5.12E-06	0.0777		
HI	14YN	Bn-scaff_15763_1-p506136	C06	20078539	2.45E-06	0.0851		
HI	14YN-CQ	Bn-scaff_15763_1-p506136	C06	20078539	1.81E-06	0.0925		
HI	13CQ	Bn-scaff_21276_1-p345305	C09	35841450	4.70E-06	0.0539	AA	GG
ST	13CQ	Bn-A01-p1745099	A01	1290703	1.80E-05	0.0509	GG	AA
ST	13CQ	Bn-A01-p4920622	A01	4515873	1.27E-05	0.0524	AA	GG
ST	13CQ	Bn-A02-p7520783	A02	4541027	1.41E-05	0.0519	TT	CC
ST	13CQ	Bn-A02-p20762322	A02	19237258	2.13E-06	0.0602	CC	TT
ST	13CQ	Bn-A02-p22798107	A02	20535892	4.96E-06	0.0565	CC	TT
ST	13CQ	Bn-A03-p4256940	A03	3794804	1.96E-06	0.0606		
ST	13CQ	Bn-A03-p4809473	A03	4295003	4.57E-07	0.0671	TT	CC
ST	13CQ	Bn-A03-p7643795	A03	6944642	1.78E-06	0.0611	AA	GG

ST	13CQ	Bn-A03-p7688578	A03	6987171	4.00E-06	0.0575	GG	AA
ST	13CQ	Bn-A03-p8475614	A03	7780068	1.18E-05	0.0527	TT	GG
ST	14YN	Bn-A03-p8822734	A03	8127204	1.95E-05	0.0739	TT	CC
ST	13CQ	Bn-A03-p9129107	A03	8414874	4.84E-06	0.0566	AA	GG
ST	13CQ	Bn-A03-p13440035	A03	12558880	1.18E-05	0.0527	CC	AA
ST	13CQ	Bn-A03-p15675871	A03	14735140	6.22E-06	0.0555	CC	TT
ST	13CQ	Bn-A03-p18376493	A03	17376477	6.68E-06	0.0552	AA	CC
ST	13CQ	Bn-A03-p19129006	A03	18067901	3.27E-06	0.0584	TT	CC
ST	13CQ	Bn-A03-p19753470	A03	18718445	7.97E-06	0.0544	GG	AA
ST	13CQ	Bn-A03-p20375514	A03	19247585	5.82E-10	0.0969	AA	GG
ST	13CQ	Bn-A03-p20409233	A03	19275298	5.54E-06	0.056	TT	CC
ST	14CQ	Bn-A03-p20662048	A03	19487581	2.94E-07	0.0643	CC	AA
ST	14CQ	Bn-A03-p20678223	A03	19502657	3.14E-05	0.0451	GG	AA
ST	14CQ	Bn-A03-p20691359	A03	19510293	9.11E-06	0.0502	CC	TT
ST	13CQ	Bn-A03-p21904842	A03	20689460	1.09E-06	0.0632	AA	GG
ST	13CQ	Bn-A04-p2222852	A04	1752487	3.12E-07	0.0687	GG	AA
ST	13CQ	Bn-A04-p2353879	A04	2069285	2.86E-07	0.0691	GG	AA
ST	13CQ	Bn-A04-p4150340	A04	4203005	1.98E-05	0.0504	TT	GG
ST	13CQ	Bn-A04-p12759499	A04	13429641	2.14E-07	0.0704	CC	TT
ST	13CQ	Bn-A04-p13050240	A04	13737904	3.00E-05	0.0486	CC	AA
ST	13CQ	Bn-A04-p15108913	A04	15545719	6.00E-06	0.0557	AA	GG
ST	13CQ	Bn-A05-p619336	A05	737605	2.21E-05	0.05	AA	GG
ST	13CQ	Bn-A05-p822597	A05	958179	1.28E-06	0.0625	AA	TT
ST	13CQ	Bn-A05-p1311652	A05	1426793	9.16E-06	0.0538	AA	CC
ST	13CQ	Bn-A05-p4516526	A05	4373236	2.59E-05	0.0492	TT	CC
ST	13CQ	Bn-A05-p6542040	A05	6205280	2.66E-05	0.0491	GG	AA
ST	13CQ	Bn-A05-p11812178	A05	10346549	2.61E-05	0.0492	TT	CC
ST	13CQ	Bn-A05-p11818095	A05	10352600	1.05E-05	0.0532	TT	CC
ST	13CQ	Bn-A05-p17708970	A05	15861727	2.04E-05	0.0503	AA	GG
ST	13CQ	Bn-A06-p3496759	A06	3282872	1.01E-05	0.0534	AA	GG
ST	13CQ	Bn-A06-p6437068	A06	5876476	8.05E-06	0.0544	AA	GG
ST	13CQ	Bn-A06-p8080390	A06	7723584	1.17E-05	0.0528	AA	GG
ST	13CQ	Bn-A06-p9299877	A06	8771431	1.96E-06	0.0606	CC	TT
ST	13CQ	Bn-A06-p11919396	A06	14582267	1.72E-06	0.0612	CC	TT
ST	13CQ	Bn-A06-p14576278	A06	16129554	8.06E-06	0.0544	TT	GG
ST	13CQ	Bn-Scaffold000289-p27459	A06	17670030	1.80E-05	0.0508	GG	AA
ST	13CQ	Bn-A06-p16406113	A06	17919297	3.60E-06	0.0579	GG	TT
ST	13CQ	Bn-A06-p16994210	A06	18408096	2.29E-08	0.0804	TT	CC
ST	13CQ	Bn-A06-p21490506	A06	20212146	3.37E-07	0.0684	AA	GG
ST	13CQ	Bn-A06-p24501540	A06	23444533	8.51E-06	0.0541	AA	CC
ST	13CQ	Bn-A06-p24695860	A06	23595420	5.19E-06	0.0563	GG	AA
ST	13CQ	Bn-A07-p1196596	A07	887590	2.79E-05	0.0489	CC	TT

ST	13CQ	Bn-scaff_17708_1-p169681	A07	1250011	5.85E-06	0.0558	AA	TT
ST	13CQ	Bn-A02-p847638	A07	13478767	1.49E-05	0.0517	AA	CC
ST	13CQ	Bn-A07-p13639374	A07	15631838	1.66E-05	0.0512	TT	CC
ST	13CQ	Bn-scaff_15818_1-p2633791	A07	15988008	2.33E-06	0.0599	GG	TT
ST	13CQ	Bn-A07-p14958606	A07	16830449	1.16E-06	0.0629		
ST	13CQ	Bn-A07-p20935217	A07	22517611	6.41E-07	0.0656	AA	GG
ST	13CQ	Bn-A08-p1068698	A08	984273	8.92E-07	0.0641	AA	GG
ST	13CQ	Bn-A08-p3031902	A08	2513455	9.89E-06	0.0535	AA	GG
ST	14VTL	Bn-A08-p3573966	A08	2951512	1.46E-05	0.0813	AA	GG
ST	14VTL	Bn-A08-p4107482	A08	3512367	1.40E-05	0.0802	TT	GG
ST	13CQ	Bn-A08-p4293226	A08	3694293	1.54E-06	0.0617	TT	CC
ST	13CQ	Bn-A08-p7814328	A08	6786884	8.00E-06	0.0544	AA	GG
ST	13CQ	Bn-A08-p8543675	A08	7389541	1.01E-06	0.0636	CC	TT
ST	13CQ	Bn-scaff_27039_1-p197834	A08	7397046	3.17E-07	0.0687	CC	TT
ST	13CQ	Bn-A08-p8634610	A08	7505807	1.77E-05	0.0509	AA	GG
ST	13CQ	Bn-A08-p11329864	A08	9184409	9.21E-06	0.0538	TT	CC
ST	13CQ	Bn-A08-p12360518	A08	10182282	1.41E-05	0.0519	AA	GG
ST	13CQ	Bn-A08-p12556455	A08	10335312	2.50E-05	0.0494	CC	TT
ST	13CQ	Bn-A08-p12752628	A08	10449877	6.40E-07	0.0656	AA	GG
ST	13CQ	Bn-A08-p13376746	A08	11121237	1.37E-05	0.0521	AA	GG
ST	13CQ	Bn-A08-p16115851	A08	13575343	2.05E-05	0.0503	AA	GG
ST	13CQ	Bn-A08-p17193797	A08	14679373	1.07E-07	0.0668		
ST	13CQ	Bn-A08-p17461463	A08	14922078	1.72E-05	0.051	CC	TT
ST	13CQ	Bn-A08-p17565873	A08	15018415	6.70E-06	0.0552	CC	TT
ST	13CQ	Bn-A08-p19676653	A08	17007108	6.50E-07	0.0655	CC	TT
ST	13CQ	Bn-A08-p20354609	A08	17822431	7.31E-06	0.0548		
ST	13CQ	Bn-A09-p1756759	A09	2180009	3.38E-07	0.0684	CC	TT
ST	13CQ	Bn-A09-p10306216	A09	9527218	9.67E-08	0.074	CC	TT
ST	13CQ	Bn-Scaffold000185-p153095	A09	16498800	3.37E-06	0.0582	AA	GG
ST	13CQ	Bn-A09-p21224256	A09	18562826	1.62E-07	0.0717	TT	CC
ST	13CQ	Bn-A07-p9535820	A09	19578645	2.97E-05	0.0486	CC	AA
ST	13CQ	Bn-A09-p33694154	A09	30983144	5.14E-06	0.0564	CC	TT
ST	14VTL	Bn-A10-p6371190	A10	7194725	1.85E-05	0.0781	GG	TT
ST	13CQ	Bn-A10-p7410271	A10	9082874	1.23E-05	0.0525	TT	CC
ST	13CQ	Bn-scaff_17750_1-p1168839	A10	15681332	6.89E-06	0.0551	AA	GG
ST	13CQ	Bn-scaff_17827_1-p271972	C01	7529799	1.74E-07	0.0713	GG	AA
ST	13CQ	Bn-scaff_22790_1-p182208	C01	15857427	3.90E-06	0.0576	AA	CC
ST	13CQ	Bn-scaff_22790_1-p370880	C01	30305788	5.00E-06	0.0565	TT	CC
ST	13CQ	Bn-scaff_22146_1-p14355	C01	34428613	1.60E-05	0.0454		

ST	13CQ	Bn-scaff_18507_1-p1252081	C02	26991241	2.80E-06	0.059	TT	CC
ST	13CQ	Bn-C13594816-p86	C02	40111275	6.40E-06	0.0554	AA	GG
ST	13CQ	Bn-scaff_17623_1-p1013272	C02	42483650	4.43E-06	0.057	CC	TT
ST	13CQ	Bn-scaff_22728_1-p360792	C03	6151071	2.60E-05	0.0492	TT	CC
ST	13CQ	Bn-scaff_18322_1-p918195	C03	8116755	2.10E-08	0.0808	AA	TT
ST	13CQ	Bn-scaff_16002_1-p1569056	C03	12839150	1.01E-06	0.0636	GG	TT
ST	13CQ	Bn-scaff_18482_1-p437945	C03	19927672	8.16E-06	0.0543	TT	CC
ST	13CQ	Bn-scaff_17521_1-p807610	C03	21410571	9.60E-07	0.0638	TT	CC
ST	13CQ	Bn-scaff_17440_1-p761980	C03	47656790	7.53E-07	0.0649	GG	AA
ST	13CQ	Bn-scaff_16182_1-p319123	C03	51969206	2.55E-06	0.0595	CC	TT
ST	13CQ	Bn-scaff_15703_1-p53309	C03	52357539	1.82E-07	0.0712	CC	AA
ST	13CQ	Bn-A08-p293480	C03	60268012	7.50E-06	0.0547	AA	GG
ST	13CQ	Bn-scaff_17869_1-p110378	C04	9129425	1.93E-05	0.0505	AA	CC
ST	13CQ	Bn-scaff_18712_1-p602624	C04	14356066	5.09E-07	0.0666	GG	TT
ST	13CQ	Bn-scaff_19253_1-p197419	C04	15181215	1.21E-05	0.0526	GG	AA
ST	13CQ	Bn-scaff_19049_1-p163830	C04	27480077	1.82E-07	0.0645	CC	AA
ST	13CQ	Bn-scaff_26787_1-p30400	C04	36649009	1.20E-05	0.0526	AA	CC
ST	13CQ	Bn-A04-p15456188	C04	44583867	5.50E-06	0.0499		
ST	13CQ	Bn-scaff_16414_1-p650418	C05	1279537	2.34E-05	0.0497	TT	CC
ST	13CQ	Bn-scaff_20901_1-p58670	C05	4042723	2.24E-05	0.0499	GG	AA
ST	13CQ	Bn-scaff_21884_1-p235276	C05	4305731	9.68E-06	0.0536	GG	AA
ST	13CQ	Bn-scaff_18181_1-p573554	C05	7093460	1.84E-05	0.0508	AA	GG
ST	13CQ	Bn-scaff_21338_1-p1197568	C05	12577310	1.24E-05	0.0525	AA	TT
ST	13CQ	Bn-scaff_16770_1-p684639	C05	35242768	8.03E-07	0.0581		
ST	14YN	Bn-scaff_15763_1-p506136	C06	20078539	7.75E-06	0.0851		
ST	13CQ	Bn-scaff_18807_1-p154933	C06	31059927	1.71E-05	0.0511	CC	GG
ST	13CQ	Bn-scaff_23771_1-p23757	C07	7037444	3.34E-06	0.0583	AA	CC
ST	13CQ	Bn-scaff_22310_1-p243575	C07	7893119	6.79E-07	0.0653	GG	TT
ST	13CQ	Bn-scaff_22310_1-p815532	C07	8399188	1.12E-05	0.0529	AA	CC
ST	13CQ	Bn-scaff_18202_1-p136363	C07	23424455	1.30E-05	0.0523	AA	GG
ST	13CQ	Bn-scaff_18181_1-p1849246	C07	34322840	5.99E-07	0.0659	CC	TT
ST	13CQ	Bn-scaff_16110_1-p1990416	C07	42889038	1.38E-06	0.0622	GG	AA
ST	13CQ	Bn-scaff_23765_1-p208	C08	7612606	3.11E-05	0.0485	AA	CC
ST	13CQ	Bn-scaff_16766_1-p179061	C08	9447598	5.82E-07	0.0595		
ST	13CQ	Bn-scaff_18602_1-p278628	C08	16438368	1.10E-07	0.0734	CC	AA
ST	13CQ	Bn-scaff_21786_1-p111311	C08	19631438	1.38E-05	0.052	CC	AA

ST	13CQ	Bn-scaff_16197_1-p1970599	C08	32209089	2.73E-07	0.0693	CC	TT
ST	13CQ	Bn-scaff_16445_1-p968765	C08	35812498	8.10E-08	0.0747	GG	AA
ST	13CQ	Bn-scaff_16456_1-p408264	C09	994523	5.43E-06	0.05		
ST	13CQ	Bn-scaff_17799_1-p3050043	C09	39518746	3.02E-05	0.0486	CC	TT
ST	13CQ	Bn-scaff_17088_1-p327069	C09	40117533	4.28E-06	0.0572	AA	GG
ST	13CQ	Bn-scaff_22082_1-p272234	C09	44582838	5.42E-07	0.0663	TT	CC
SY	13CQ	Bn-A02-p10067386	A02	6895619	2.61E-05	0.0538	CC	TT
SY	14VTL	Bn-A03-p11544253	A03	10669935	1.23E-05	0.0852	GG	AA
SY	14CQ	Bn-A03-p19727558	A03	18670128	2.42E-05	0.0424	AA	CC
SY	13CQ	Bn-A03-p24017859	A03	22660395	1.50E-05	0.0565		
SY	14VTL	Bn-scaff_16517_1-p192158	A04	14284133	3.20E-06	0.0848		
SY	14YN-CQ	Bn-scaff_16517_1-p192158	A04	14284133	6.86E-06	0.0812		
SY	14VTL	Bn-A04-p17623914	A04	17733410	2.66E-05	0.0796	TT	CC
SY	13CQ	Bn-A05-p701389	A05	815813	2.37E-05	0.0543	CC	AA
SY	14VTL	Bn-A06-p22947499	A06	21994293	1.02E-05	0.0866	TT	CC
SY	14VTL	Bn-A06-p23865356	A06	22856804	4.14E-07	0.1101	CC	TT
SY	14VTL	Bn-A06-p23873505	A06	22863780	1.32E-05	0.0847	AA	GG
SY	13CQ	Bn-A09-p25738298	A09	23823306	1.53E-05	0.0564	CC	TT
SY	13CQ	Bn-A09-p5118197	A09	29701178	3.30E-07	0.0749	CC	TT
SY	13CQ	Bn-scaff_19526_1-p67	C01	16351149	5.02E-06	0.0617	AA	GG
SY	14CQ	Bn-scaff_19170_1-p1132242	C04	18833673	2.35E-05	0.0425	TT	GG
SY	13CQ	Bn-scaff_20270_1-p1110017	C05	41579418	1.55E-05	0.0563	TT	GG
SY	13CQ	Bn-scaff_16547_1-p307962	C06	29728738	1.13E-05	0.0579	TT	CC
SY	13CQ	Bn-scaff_16197_1-p974627	C08	33120770	5.33E-06	0.0615	AA	GG
SY	13CQ	Bn-scaff_16197_1-p974284	C08	33121140	7.28E-06	0.06	TT	GG

Supplementary Table S3. Partial SNPs and candidate genes with pleiotropic effects.

SNP	Chr.	Position (bp)	Associated traits	Allele	MAF	LD interval (kb)	List of candidate genes [a]	HI-related DEGs or TFs [b]
Bn-A02-p10067386	A02	6895619	13CQ-HI, 13CQ-SY	C/T	0.128	376	BnaA02g12550D~BnaA02g13050D	BnaA02g12850D (LBD40)
								BnaA02g13010D (TCP1)
								BnaA02g12860D (CYP735A2)
Bn-A03-p20375514	A03	19247585	13CQ-CBY, 13CQ-ST, 13CQ-BY	A/G	0.325	498	BnaA03g38390D~BnaA03g39260D	BnaA03g38390D (NF-YB7)
								BnaA03g38660D (VSR3)
Bn-scaff_16517_1-p192158	A04	14284133	14VTL-SY, 14YN-CQ-SY	C/A	0.082	486	BnaA04g16920D~BnaA04g17640D	BnaA04g17560D (C4H)
Bn-A06-p23865356	A06	22856804	14VTL-HI, 14VTL-SY, 14YN-CQ-HI	C/T	0.048	289	BnaA06g34100D~BnaA06g34630D	BnaA06g34140D (LOV1)
								BnaA06g34390D (SGR5)
Bn-A06-p23873505	A06	22863780	14VTL-SY, 14YN-CQ-HI	A/G	0.097	289	BnaA06g34100D~BnaA06g34630D	
Bn-A06-p24501540	A06	23444533	13CQ-CBY, 13CQ-ST, 13CQ-BY	A/C	0.41	231	BnaA06g35190D~BnaA06g35710D	BnaA06g35340D(NF-YB6)
Bn-scaff_17708_1-p169681	A07	1250011	13CQ-CBY, 13CQ-ST, 13CQ-BY	T/A	0.431	317	BnaA07g01290D~BnaA07g01630D	BnaA07g01580D
								BnaA07g01410D (ZIF2)
Bn-A08-p7814328	A08	6786884	13CQ-CBY, 13CQ-ST, 13CQ-BY	G/A	0.444		BnaA08g06680D~BnaA08g06940D	BnaA08g06940D (RCD1)
Bn-A08-p8543675	A08	7389541	13CQ-CBY, 13CQ-ST, 13CQ-BY	T/C	0.359	408	BnaA08g07300D~BnaA08g07870D	BnaA08g06730D (PRPL11)
								BnaA08g06910D (HSFA1D)
Bn-A08-p12752628	A08	10449877	13CQ-CBY, 13CQ-ST, 13CQ-BY	A/G	0.332		BnaA08g11410D~BnaA08g11710D	BnaA08g11640D (CCoAOMT1)
Bn-A08-p20354609	A08	17822431	13CQ-CBY, 13CQ-ST, 13CQ-BY	C/T	0.353	490	BnaA08g26110D~BnaA08g27380D	BnaA08g26150D (CDC5)
								BnaA08g26320D(MYB61)
								BnaA08g26390D (ACLA-3)

Bn-A09-p33694154	A09	30983144	13CQ-CBY, 13CQ-ST, 13CQ-BY	T/C	0.361	472	BnaA09g44990D~BnaA09g45920D	BnaA09g45190D (CYP72C1)<br>BnaA09g45200D (SRG1)<br>BnaA09g45470D (KNOX1)
Bn-scaff_18482_1-p437945	C03	19927672	13CQ-CBY, 13CQ-ST, 13CQ-BY	C/T	0.341	276	BnaC03g31950D~BnaC03g32440D	BnaC03g32200D (ROXY1)
Bn-scaff_17521_1-p807610	C03	21410571	13CQ-CBY, 13CQ-ST, 13CQ-BY	T/C	0.428	309	BnaC03g35210D~BnaC03g35790D	BnaC03g35240D (AIR12)<br>BnaC03g35440D (COL9)
Bn-A08-p293480	C03	60268012	13CQ-CBY, 13CQ-ST, 13CQ-BY	G/A	0.263		BnaC03g70510D~BnaC03g70770D	BnaC03g70660D (WRKY10)
Bn-scaff_16770_1-p684639	C05	35242768	13CQ-CBY, 13CQ-ST, 13CQ-BY	C/A	0.255		BnaC05g35970D~BnaC05g36190D	BnaC05g36130D (DAYSLEEPER)
Bn-scaff_15763_1-p506136	C06	20078539	14YN-HI, 14YN-ST, 14VTL-HI, 14YN-CQ-HI	G/T	0.175	290	BnaC06g17100D~BnaC06g17510D	BnaC06g17400D (LRR)
Bn-scaff_18181_1-p1849246	C07	34322840	13CQ-CBY, 13CQ-ST, 13CQ-BY	T/C	0.334		BnaC07g29030D~BnaC07g30110D	BnaC07g29270D<br>BnaC07g29460D (SPS2F)
Bn-scaff_23765_1-p208	C08	7612606	13CQ-CBY, 13CQ-ST, 13CQ-BY	A/C	0.361		BnaC08g05480D~BnaC08g05670D	BnaC08g05510D
Bn-scaff_18602_1-p278628	C08	16438368	13CQ-CBY, 13CQ-ST, 13CQ-BY	A/C	0.313		BnaC08g10880D~BnaC08g11100D	BnaC08g10960D (HDG4)
Bn-scaff_16197_1-p1970599	C08	32209089	13CQ-CBY, 13CQ-ST, 13CQ-BY	C/T	0.408	479	BnaC08g33390D~BnaC08g34150D	BnaC08g33510D (F3H)<br>BnaC08g33690D (CDD1)

[a] List of candidate genes were obtained from *B. napus* gene annotation in the LD intervals.

[b] All the important candidate DEGs and TFs were annotated by BLASTP analysis. The corresponding protein names were in the brack

Supplementary Table S4. Read mapping summary for each sample in *B. napus*.

Sample [a]	Number of input reads	Uniquely mapped reads number	Uniquely mapped reads %	Number of splices: Total	Mismatch rate per base, %	Deletion rate per base	Deletion average length	Insertion rate per base	Insertion average length	Number of reads mapped to multiple loci	% of reads mapped to multiple loci	Number of reads mapped to too many loci	% of reads mapped to too many loci	% of reads unmapped: other
cBP-H1	22452858	18864487	84.02%	13476963	0.58%	0.03%	2.67	0.02%	2.34	1792737	7.98%	1016958	4.53%	0.02%
cBP-H2	23514750	19485491	82.86%	14005727	0.61%	0.03%	2.7	0.02%	2.3	1892137	8.05%	1230900	5.23%	0.02%
cBP-L1	28303470	22914737	80.96%	16365855	0.58%	0.03%	2.57	0.02%	2.33	2270471	8.02%	2184646	7.72%	0.02%
cBP-L2	24738173	19601236	79.23%	13691861	0.58%	0.03%	2.55	0.02%	2.27	2004687	8.10%	2251575	9.10%	0.02%
cBM-H1	24364033	19384121	79.56%	13845181	0.59%	0.03%	2.69	0.02%	2.33	2004247	8.23%	2176135	8.93%	0.02%
cBM-H2	20976417	17325240	82.59%	12466949	0.62%	0.03%	2.65	0.02%	2.35	1680094	8.01%	1284533	6.12%	0.02%
cBM-L1	22536528	18257644	81.01%	13272192	0.58%	0.03%	2.6	0.02%	2.32	1802195	8.00%	1724629	7.65%	0.02%
cBM-L2	21614555	17139602	79.30%	12339510	0.58%	0.03%	2.59	0.02%	2.35	1753799	8.11%	1961795	9.08%	0.02%
cLe-H1	16489851	14099181	85.50%	10259121	0.54%	0.03%	2.67	0.02%	2.38	1441373	8.74%	453243	2.75%	0.02%
cLe-H2	20231639	17344269	85.73%	12664647	0.55%	0.03%	2.71	0.02%	2.37	1707942	8.44%	506333	2.50%	0.02%
cLe-L1	22845808	18027345	78.91%	12829722	0.57%	0.03%	2.51	0.02%	2.39	2594154	11.36%	1477234	6.47%	0.02%
cLe-L2	15105088	12103042	80.13%	8913829	0.53%	0.03%	2.72	0.02%	2.42	1663767	11.01%	906620	6.00%	0.01%
cSP-H1	23181999	18927871	81.65%	10667165	0.55%	0.03%	2.46	0.02%	2.38	2035543	8.78%	1362750	5.88%	0.01%
cSP-H2	23899337	19802639	82.86%	11026884	0.55%	0.03%	2.43	0.02%	2.4	2080906	8.71%	1018105	4.26%	0.01%
cSP-L1	27821443	22279420	80.08%	12866951	0.53%	0.03%	2.51	0.02%	2.38	2452974	8.82%	2093498	7.52%	0.02%
cSP-L2	24730497	19366948	78.31%	11176988	0.55%	0.02%	2.39	0.02%	2.39	2131942	8.62%	2339752	9.46%	0.01%
cSM-H1	20538834	15964938	77.73%	9147573	0.56%	0.03%	2.47	0.02%	2.35	1800764	8.77%	1973128	9.61%	0.02%
cSM-H2	23634762	19510271	82.55%	11508656	0.56%	0.03%	2.4	0.02%	2.36	2079305	8.80%	1107745	4.69%	0.02%

cSM-L1	22939882	18126271	79.02%	10050144	0.52%	0.03%	2.48	0.02%	2.37	1982443	8.64%	2035975	8.88%	0.02%
cSM-L2	22902919	18522381	80.87%	10358975	0.53%	0.03%	2.44	0.02%	2.37	2060086	8.99%	1504922	6.57%	0.02%
cSPP-H1	22151383	17418557	78.63%	12792969	0.60%	0.03%	2.63	0.02%	2.36	1970437	8.90%	1786356	8.06%	0.03%
cSPP-H2	20247972	16986266	83.89%	12461172	0.60%	0.03%	2.63	0.02%	2.35	1589953	7.85%	839639	4.15%	0.03%
cSPP-L1	24903583	20620948	82.80%	14929227	0.57%	0.03%	2.62	0.02%	2.38	2013042	8.08%	1287507	5.17%	0.03%
cSPP-L2	29944775	25488721	85.12%	18202479	0.56%	0.03%	2.61	0.02%	2.37	2295167	7.66%	1037009	3.46%	0.03%
cSPM-H1	21408332	17357702	81.08%	12816796	0.60%	0.03%	2.63	0.02%	2.36	1858109	8.68%	1291707	6.03%	0.03%
cSPM-H2	16910482	14841728	87.77%	11291600	0.55%	0.03%	2.78	0.02%	2.48	1137840	6.73%	298611	1.77%	0.03%
cSPM-L1	21311302	18522700	86.91%	13646999	0.56%	0.03%	2.62	0.02%	2.38	1567372	7.35%	449130	2.11%	0.03%
cSPM-L2	16808020	14889727	88.59%	11100330	0.53%	0.03%	2.76	0.02%	2.42	1116796	6.64%	248129	1.48%	0.03%
cSt-H1	25596645	20451155	79.90%	13859010	0.58%	0.03%	2.6	0.02%	2.37	2149692	8.40%	2162286	8.45%	0.02%
cSt-H2	23638567	19034548	80.52%	12854480	0.61%	0.03%	2.64	0.02%	2.39	1911822	8.09%	1972012	8.34%	0.03%
cSt-L1	19737745	16449191	83.34%	11287265	0.59%	0.03%	2.65	0.02%	2.31	1423172	7.21%	1116432	5.66%	0.03%
cSt-L2	22468133	18687369	83.17%	12945024	0.55%	0.03%	2.6	0.02%	2.27	1625567	7.23%	1290177	5.74%	0.03%
yBP-H1	18754043	16119980	85.95%	11949161	0.62%	0.03%	2.67	0.02%	2.28	1411407	7.53%	414208	2.21%	0.02%
yBP-H2	23958075	20503988	85.58%	15130377	0.64%	0.03%	2.72	0.02%	2.31	1712551	7.15%	555643	2.32%	0.02%
yBP-L1	29073659	24826331	85.39%	18080792	0.60%	0.03%	2.66	0.02%	2.33	2205289	7.59%	640707	2.20%	0.02%
yBP-L2	23906053	20100960	84.08%	14395280	0.62%	0.03%	2.63	0.02%	2.33	1825074	7.63%	941027	3.94%	0.02%
yBM-H1	17270196	14836580	85.91%	10952836	0.62%	0.03%	2.69	0.02%	2.24	1278456	7.40%	372621	2.16%	0.02%
yBM-H2	22129356	18883643	85.33%	14181017	0.55%	0.03%	2.73	0.02%	2.39	1616954	7.31%	800354	3.62%	0.02%
yBM-L1	25621566	21677778	84.61%	15543034	0.62%	0.03%	2.69	0.02%	2.33	1982507	7.74%	807283	3.15%	0.03%
yBM-L2	22994851	19019184	82.71%	13587026	0.62%	0.03%	2.66	0.02%	2.34	1742710	7.58%	742626	3.23%	0.02%
yLe-H1	19140351	16249757	84.90%	12099935	0.56%	0.03%	2.65	0.02%	2.26	1549791	8.10%	369068	1.93%	0.03%
yLe-H2	17229947	14664605	85.11%	10900732	0.56%	0.03%	2.68	0.02%	2.25	1360496	7.90%	264391	1.53%	0.03%
yLe-L1	19475952	16041270	82.36%	11923206	0.60%	0.03%	2.62	0.02%	2.31	1933970	9.93%	690708	3.55%	0.03%

yLe-L2	19553720	16636230	85.08%	12126744	0.59%	0.03%	2.63	0.02%	2.38	1732009	8.86%	389793	1.99%	0.03%
ySP-H1	20267060	17141854	84.58%	9881663	0.58%	0.02%	2.51	0.02%	2.27	1832213	9.04%	415275	2.05%	0.02%
ySP-H2	15346963	12918966	84.18%	7619628	0.58%	0.02%	2.46	0.02%	2.26	1352763	8.81%	402271	2.62%	0.02%
ySP-L1	27061541	22209895	82.07%	12572158	0.55%	0.02%	2.45	0.02%	2.31	2481543	9.17%	1108675	4.10%	0.02%
ySP-L2	22387495	19043081	85.06%	11739477	0.54%	0.03%	2.51	0.02%	2.29	1901962	8.50%	442584	1.98%	0.02%
ySM-H1	20025596	16747744	83.63%	10018027	0.58%	0.03%	2.44	0.02%	2.22	1749319	8.74%	501603	2.50%	0.02%
ySM-H2	32535971	27668883	85.04%	16361125	0.59%	0.03%	2.51	0.02%	2.24	2838703	8.72%	607201	1.87%	0.02%
ySM-L1	29955765	24690799	82.42%	13531485	0.54%	0.02%	2.4	0.02%	2.34	2879027	9.61%	1049638	3.50%	0.02%
ySM-L2	18563211	15522984	83.62%	8879260	0.53%	0.02%	2.56	0.02%	2.45	1674212	9.02%	678307	3.65%	0.01%
ySPP-H1	14944658	13089842	87.59%	9681553	0.62%	0.03%	2.68	0.02%	2.33	1014695	6.79%	135844	0.91%	0.03%
ySPP-H2	15403148	13507038	87.69%	10118084	0.62%	0.03%	2.65	0.02%	2.27	1026736	6.67%	148106	0.96%	0.03%
ySPP-L1	24034024	20843093	86.72%	15010760	0.55%	0.03%	2.62	0.02%	2.35	1718350	7.15%	180384	0.75%	0.03%
ySPP-L2	19321573	16718027	86.53%	11735296	0.61%	0.03%	2.59	0.02%	2.3	1454533	7.53%	219043	1.13%	0.03%
ySPM-H1	15043615	13155753	87.45%	9892032	0.61%	0.03%	2.68	0.02%	2.31	1024244	6.81%	158111	1.05%	0.04%
ySPM-H2	17076382	14782172	86.57%	9507820	0.52%	0.03%	2.63	0.02%	2.42	1343418	7.87%	382746	2.24%	0.02%
ySPM-L1	19783193	17076386	86.32%	12509635	0.56%	0.03%	2.66	0.02%	2.35	1368420	6.92%	176671	0.89%	0.03%
ySPM-L2	23216716	20228298	87.13%	14731839	0.55%	0.03%	2.63	0.02%	2.35	1616485	6.96%	122266	0.53%	0.03%
ySt-H1	21465254	18723577	87.23%	13577566	0.56%	0.03%	2.67	0.02%	2.41	1400726	6.53%	234212	1.09%	0.03%
ySt-H2	16023517	13806135	86.16%	9981864	0.61%	0.03%	2.69	0.02%	2.35	1062960	6.63%	327378	2.04%	0.02%
ySt-L1	18418094	15885614	86.25%	11614022	0.60%	0.03%	2.79	0.02%	2.47	1243514	6.75%	565282	3.07%	0.03%
ySt-L2	17119155	14339182	83.76%	10495995	0.56%	0.03%	2.82	0.02%	2.49	1346533	7.87%	786418	4.59%	0.02%
Average	21641414	18053990.23	83.62%	12335182	0.58%	0.03%	2.60875	0.02%	2.345625	1758939	8.09%	937811	4.17%	0.02%
Total	1385050482	1155455375		789451673						112572105		60019915		

Supplementary Table S5. Summary of significantly enriched GO and KEGG terms.

Sample name	GO terms		KEGG terms	
	up-regulated	down-regulated	up-regulated	down-regulated
cSPP	252	104	6	3
cBP	166	23	4	0
cSP	158	243	2	7
cLe	51	86	1	2
cSt	382	242	7	5
cSPM	436	103	12	2
cBM	310	56	6	0
cSM	325	454	11	10
ySPP	481	372	5	9
yBP	122	0	2	0
ySP	644	173	13	3
yLe	48	252	0	3
ySt	78	309	0	6
ySPM	408	418	11	4
yBM	3	67	3	0
ySM	267	62	9	0
Total	4131	2964	92	54

First letters (c and y) in the sample names represent the cultivation region Chongqing and Yunnan, respectively. SPP: silique pericarps on the primary branch, BP: buds on the primary branch, SP: seeds harvested 20 days after flowering on the primary branch, Le: leaves, St: stems, SPM: silique pericarps on the main inflorescence, BM: buds on the main inflorescence, SM: seeds harvested 20 days after flowering on the main inflorescence.

Supplementary Table S6. Pearson's correlation coefficients for trait pairs affecting HI.

Environment	Trait	CBY	ST	BY	SY	HI
13CQ	ST	0.71**	1			
	BY	0.97**	0.86**	1		
	SY	0.61**	0.44**	0.60**	1	
	HI	0.36**	0.15	0.31**	0.66**	1
14CQ	ST	0.58**	1			
	BY	0.92**	0.84**	1		
	SY	0.85**	0.51**	0.79**	1	
	HI	0.40**	−0.04	0.25**	0.72**	1
14YN	ST	0.29**	1			
	BY	.082**	0.72**	1		
	SY	0.79**	-0.06	0.51**	1	
	HI	0.46**	−0.48**	0.08	0.84**	1
14YN-CQ	ST	0.38**	1			
	BY	0.86**	0.74**	1		
	SY	0.80**	0.09	.592**	1	
	HI	0.47**	−0.31**	0.17**	0.82**	1
14VTL	ST	0.46**	1			
	BY	0.90**	0.77**	1		
	SY	0.80**	0.21**	0.64**	1	
	HI	0.52**	−0.20**	0.26**	0.86**	1

*$p < 0.05$, **$p < 0.01$, respectively.

Supplementary Table S7. List of extremely high- and low-HI *B. napus* accessions included in the RNA-seq analysis.

Cultivated region	Group	Accession number	Accession name	Type	Provider
YN	high HI	B376	Zhongshuang12	Semi-winter	ORI, CAAS
		B330	SWU41	Semi-winter	SWU
		B122	2012-8380	Semi-winter	ORI, CAAS
		B189	Huashuang128	Semi-winter	HZAU
	low HI	B341	SWU68	Semi-winter	SWU
		B400	Zhongshuang4	Semi-winter	ORI, CAAS
		B416	FY9-1	Semi-winter	HAAS
		B163	09-P64-1	Semi-winter	HAZU
CQ	high HI	B400	Zhongshuang4	Semi-winter	ORI, CAAS
		B18	SWU47	Semi-winter	SWU
		B25	SWU82	Semi-winter	SWU
		B30	SWU108	Semi-winter	SWU
	low HI	B376	Zhongshuang12	Semi-winter	ORI, CAAS
		B416	FY9-1	Semi-winter	HAAS
		B56	wx10315	Semi-winter	HNAU
		B163	09-P64-1	Semi-winter	HAZU

HAAS, Henan Academy of Agricultural Sciences; HAZU, Huazhong Agricultural University; HNZU, Hunan Agricultural University; ORI, CAAS, Oilcrop Research Institute, Chinese Academy of Agricultural Sciences; SWU, Southwest University

Supplementary Table S8. Primers used for qRT-PCR analysis.

Gene name	Primer sequence (5′→ 3′)	Tm (°C)	AT (°C)	PLC (bp)
BnaA01g00480D	F: GTTAGAAACGGCAGTGTGGTGG	61.9	58	73
	R: CTATACACGCACGGACACAACG	61.8		
BnaA04g02500D	F: GCGCGGAAGATTCAAGGATGAG	61.9	58	153
	R: TTCGGTTGCAACAGAGCGAATC	62		
BnaC03g14930D	F: CCGAGAAGAGCAAAGAAGATCCA	60.4	58	144
	R: GCTCTCAGTAAGTCTTCTAGGTCAA	59.6		
BnaC03g04070D	F: GCTCAAATACTGGACAACATTCATCATCTT	62.5	58	118
	R: GGTAAACCCTGCTCAGATATTTCGG	61.5		
BnaC05g48550D	F: GCCGCCGAGATCACCAAG	60.9	58	116
	R: GCGTGTCTCCGGGGCGGA	67.1		
BnaC07g08220D	F: GGAAGCTTGGCTTGACTGGTG	61.8	58	82
	R: GCATCATCATCTACATAAAAGGAAGTAG	58.4		
BnaC07g46180D	F: TCCACGGTTACATCCAATGGCT	62	58	148
	R: AGCTAGTACCTCCCGGTCAAGA	62		
BnaC08g25790D	F: GGACAAGAAGGCATGCGACAATG	62.8	58	121
	R: GCCTCGTTTGCCCGTGTATG	62		
BnaC08g33690D	F: CCGACGCTATATATGTCCCGGG	62.1	58	173
	R: AAAACCATATGGGACCCGGTGT	62		
BnaCnng47940D	F: GTCGACCGTGGAAGCAGTACT	62.1	58	148
	R: GACCCAGGACCCGAATTACCAA	62		
Bna.ACT7	F: TGGGTTTGCTGGTGACGAT	59.5	58	63
	R: TGCCTAGGACGACCAACAATACT	61.4		
Bna.UBC21	F: CCTCTGCAGCCTCCTCAAGT	61.6	58	77
	R: CATATCTCCCCTGTCTTGAAATGC	59.5		

F: forward primer; R: reverse primer; Tm: melting temperature; AT: annealing temperature; PLC: product length of cDNA sequences.
